# Fine-tuning licensing strategies to boost MSC-based immunomodulatory secretome

**DOI:** 10.1186/s13287-025-04315-4

**Published:** 2025-04-17

**Authors:** Maria Rossello-Gelabert, Manoli Igartua, Edorta Santos-Vizcaino, Rosa Maria Hernandez

**Affiliations:** 1 NanoBioCel Research Group, Laboratory of Pharmaceutics, School of Pharmacy, University of the Basque Country (UPV/EHU), Paseo de la Universidad 7, Vitoria Gasteiz, 01006 Spain; 2 Biomedical Research Networking Centre in Bioengineering, Biomaterials and Nanomedicine (CIBER-BBN), Institute of Health Carlos III, Madrid, Spain; 3 Bioaraba, NanoBioCel Research Group, Vitoria Gasteiz, Spain

**Keywords:** Mesenchymal stromal cell (MSC), Secretome, Inflammatory licensing, Immunomodulation, Immune-mediated inflammatory diseases (IMIDs)

## Abstract

**Background:**

Immune-mediated inflammatory diseases (IMIDs) are a major global health challenge, affecting millions of people and often lacking effective treatments. The mesenchymal stromal cell (MSC)-derived secretome has emerged as a promising therapeutic approach owing to its potent immunomodulatory properties. However, progress has been hindered by the lack of standardized protocols for inducing a robust immunomodulatory MSC phenotype.

**Methods:**

In this study, we focused on optimizing the MSC-derived secretome to enhance its ability to suppress activated immune cells. Specifically, we examined (1) the effects of IFN-γ and TNF-α, individually and in combination, to uncover potential synergy; (2) the ideal cytokine ratio and (3) concentration; (4) the best production time for the secretome; and (5) the impact of cellular confluence. These factors were systematically evaluated to assess their influence on cell behavior, viability, cytosolic content release, and the secretion of key immunomodulatory and regenerative factors.

**Results:**

Our results demonstrate that overnight licensing with a 1:1 ratio of IFN-γ and TNF-α at 60 ng/mL, followed by 48 h of incubation at 90% confluence, yields an optimized conditioned media (CM) with significantly enhanced immunomodulatory properties. Functional assays showed that this CM can inhibit human peripheral blood mononuclear cell (PBMC) activation with more than twice the effectiveness of suboptimal protocols. Additionally, we found that direct cell-cell contact was critical for inducing regulatory T cells (Tregs), highlighting the complex dynamics of immune regulation.

**Conclusions:**

These findings establish a robust and standardized MSC licensing protocol, paving the way for the development of innovative and effective therapies to combat IMIDs.

**Clinical trial number:**

Not applicable.

**Supplementary Information:**

The online version contains supplementary material available at 10.1186/s13287-025-04315-4.

## Graphical Abstract

**Supplementary Information:**

The online version contains supplementary material available at 10.1186/s13287-025-04315-4.

## Graphical abstract

**Figure:**
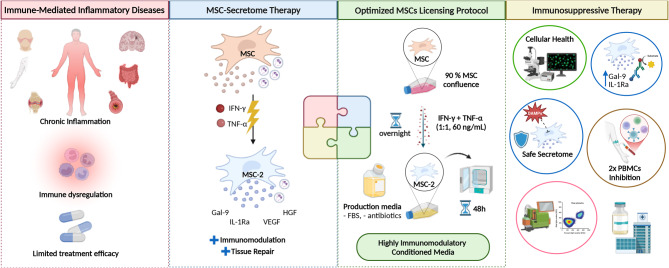

## Introduction

Immune-mediated inflammatory diseases (IMIDs) – such as rheumatoid arthritis, inflammatory bowel disease, multiple sclerosis, and systemic lupus erythematosus – comprise a diverse group of chronic conditions characterized by persistent inflammation and dysregulated immune responses [1, 2]. These diseases can affect multiple organs, either simultaneously or sequentially, resulting in significant morbidity and a reduced quality of life [3]. Collectively, IMIDs impact millions of individuals worldwide, representing a substantial global health burden [1–3].

Despite their diverse etiologies, IMIDs share common pathogenic mechanisms, primarily driven by excessive inflammation and immune dysregulation [1]. Current therapeutic approaches – including corticosteroids, immunosuppressants, small molecules, and biologics – mainly target specific inflammatory pathways. However, suboptimal therapeutic responses and treatment-related intolerance remain significant challenges, underscoring the urgent need for more effective and comprehensive immunomodulatory strategies [4–7].

In this line, emerging therapies aim to address this gap by modulating immune responses more holistically [8, 9], targeting both inflammation suppression and immune regulation [10, 11]. Among these, mesenchymal stromal cells (MSCs) have gained significant attention due to their potent immunomodulatory properties, mediated through interactions with various immune cells – including macrophages, dendritic cells, and T cells – and the secretion of bioactive molecules collectively referred to as the secretome [10, 11].

The MSC-secretome includes soluble factors such as galectin-9 (Gal-9), interleukin-1 receptor antagonist (IL-1Ra), hepatocyte growth factor (HGF), and vascular endothelial growth factor (VEGF), as well as extracellular vesicles [12, 13]. This secretome plays a central role in mitigating inflammation, promoting tissue repair, and restoring immune homeostasis, positioning it as a promising therapeutic candidate for IMIDs [1, 11]. The immunomodulatory potential of MSCs is closely linked to their immunoplasticity – the ability to shift from a pro-inflammatory (MSC1) to an anti-inflammatory (MSC2) phenotype in response to environmental cues [14–16]. The acquisition of the MSC2 phenotype is crucial for the release of immunomodulatory factors, a process driven by microenvironmental signals [11, 17, 18]. Thus, optimizing these signals is essential for enhancing the production of an immunomodulatory secretome [11, 17].

To achieve this, various licensing strategies have been explored to optimize the therapeutic efficacy of MSCs, including hypoxic, mechanical, and biochemical approaches [18]. Hypoxic licensing mimics the oxygen-deprived conditions of injured tissues, enhancing MSC survival and promoting the secretion of regenerative factors [19–21]. Mechanical licensing, on the other hand, uses physical stimuli to modulate MSC behavior and cytokine production [22]. Among these, biochemical licensing has emerged as the most effective strategy for inducing the MSC2 phenotype and generating a highly immunomodulatory secretome [18, 23–25].

Despite its potential, biochemical licensing lacks standardized protocols, leading to inconsistencies in cytokine combinations, concentrations, and secretome production times [18, 23–32]. While previous studies have explored cytokine licensing, few have systematically examined how variations in these parameters influence the quality and potency of the resulting secretome [18, 23–25, 27–30, 33–36]. Addressing these inconsistencies is crucial for improving reproducibility and clinical translation.

Within this framework, interferon-gamma (IFN-γ) and tumor necrosis factor-alpha (TNF-α) are the most commonly used cytokines due to their ability to enhance MSC immunomodulatory function [18, 23–25, 27–30, 33–36]. Exposure of MSCs to IFN-γ and TNF-α activates intracellular signaling pathways such as JAK/STAT1 (driven by IFN-γ) and nuclear factor kappa-light-chain-enhancer of activated B cells (NF-κB) (triggered by TNF-α), synergistically upregulating immunoregulatory factors including indoleamine 2,3-dioxygenase (IDO), prostaglandin E2 (PGE2), Human Leukocyte Antigen G (HLA-G), and interleukin-6 (IL-6). This cytokine-mediated “licensing” process enhances the immunosuppressive capacity of MSCs by promoting metabolic reprogramming – namely a shift toward aerobic glycolysis – and increasing the secretion of anti-inflammatory mediators. IFN-γ primes signal transducer and activator of transcription 1 (STAT1)-dependent pathways to induce expression of IDO and programmed death-ligand 1 (PD-L1), while TNF-α amplifies IFN-γ signaling through NF-κB activation and further upregulates adhesion molecules such as intercellular adhesion molecule 1 (ICAM-1) and vascular cell adhesion molecule 1 (VCAM-1), which facilitate MSC–immune cell interactions. Together, these effects suppress T cell proliferation, recruit regulatory immune cells, and modulate inflammation in a dose-dependent manner. Notably, low levels of IFN-γ may support antigen presentation via major histocompatibility complex (MHC) class II, whereas high levels drive a more immunosuppressive phenotype. Prolonged exposure, however, can induce autophagy or apoptosis, highlighting the need to balance therapeutic efficacy with MSC viability. Taken together, these mechanisms explain why IFN-γ and TNF-α are frequently used in combination to enhance MSC immunoregulatory function [23–30, 33, 34, 36].

Building on this, previous studies suggest potential synergistic effects when combining these cytokines, but the precise conditions required to maximize their immunomodulatory potency remain unclear [23–31]. Exploring both individual and combined effects of these cytokines is therefore necessary to refine licensing protocols and enhance the therapeutic outcomes.

Despite their widespread use, no consensus exists on the optimal licensing conditions. Key parameters, such as cytokine concentrations (ranging from 10 ng/mL to over 100 ng/mL), exposure times (from a few hours to several days), and specific cytokine combinations, vary significantly between studies [18, 23–31, 33–36]. Moreover, the absence of systematic quality controls to verify MSC activation contributes to inconsistencies, hindering both reproducibility and clinical translation.

To address these gaps, we systematically evaluated cytokine combinations, concentrations, and secretome collection times, aiming to establish a standardized and reproducible biochemical licensing protocol that optimizes the immunomodulatory properties of the MSC-secretome. Specifically, we examined the effects of two pro-inflammatory cytokines (IFN-γ and TNF-α), both individually and in combination, to explore potential synergistic effects. In this context, we selected immortalized human adipose tissue-derived mesenchymal stromal cells (hTERT-AT-MSCs) as our MSC model for optimization. Adipose tissue-derived MSCs (AT-MSCs) have been widely recognized for their strong immunomodulatory capacity, often surpassing bone marrow- and umbilical cord-derived MSCs in their ability to suppress peripheral blood mononuclear cells (PBMC) activation, partly due to higher expression of IDO in response to inflammatory stimuli [37]. Furthermore, the use of an hTERT-immortalized AT-MSC line ensured a homogeneous, consistent, and scalable source of secretome, minimizing donor-to-donor variability and circumventing replicative senescence – limitations commonly observed in primary MSC cultures [38–40]. This strategy allowed us to standardize experimental conditions and reliably assess the impact of cytokine licensing on the immunomodulatory properties of the MSC-derived secretome.

Additionally, we assessed the optimal cytokine ratio (1:1, 2:1, 1:2), increasing concentrations of the licensing cocktail (0–100 ng/mL), secretome production duration (24, 48, or 72 h), and the influence of cellular confluence (60–90%) on secretome composition and bioactivity.

These variables were assessed for their impact on critical parameters, including cellular behavior and viability, the release of cytosolic content into the conditioned media (CM), and changes in the secretory profile of key immunomodulatory cytokines and growth factors. Specifically, we explored how these factors influenced the production of immunomodulatory molecules (Gal-9, IL-1Ra) and growth factors (HGF, VEGF). Based on these results, we defined the optimal licensing conditions and demonstrated their efficacy in functional assays. These findings establish a standardized licensing protocol and provide a foundation for further development of MSC-based therapies into innovative clinical solutions targeting IMIDs.

## Materials and methods

### Cell culture

Immortalized human adipose tissue-derived mesenchymal stromal cells (hTERT-AT-MSCs, also known as ASC52telo, ATCC^®^ SCRC-4000) were cultured in complete MSC basal medium (ATCC^®^ PCS-500-030), supplemented with the MSC growth kit (ATCC^®^ PCS-500-040). Cells were maintained at 37 °C in a humidified atmosphere of 5% CO₂ and were passaged every 4–6 days upon reaching 60–90% confluence. All experiments were conducted using cells between passages 4 and 7 to ensure consistency in cell behavior. It should be noted that the ASC52telo (SCRC-4000) cell line has been extensively used in regenerative medicine and immunomodulatory research [41].

#### Blood sample procurement and ethical approvals

Buffy coats were obtained from healthy donors through the Basque Biobank in compliance with Law 14/2007 (Biomedical Research) and Royal Decree 1716/2011, which regulate the handling and storage of biological samples for research. The study protocol was approved by the Ethics Committee for Research Involving Human Subjects (CEISH) at the University of the Basque Country (UPV/EHU, M10_2022_131MR1). Donors provided written informed consent for the use of their blood samples. Additionally, the use and manipulation of cells were approved by the Research Ethics Committee for Biological Agents and Genetically Modified Organisms (CEIAB, M30_2022_132MR1). Peripheral blood mononuclear cells (PBMCs) were isolated from buffy coats by density gradient centrifugation and stored at -80 °C until further use.

### Conditioned media (CM) production and characterization

#### Licensed CM

To determine the optimal conditions for immunomodulatory CM production, hTERT-AT-MSCs were initially cultured to 90% confluence. The cells were then subjected to inflammatory licensing to induce the MSC2 phenotype. We first compared the effects of individual cytokines, IFN-γ and TNF-α, and their synergistic combination at an initial concentration of 20 ng/mL. Subsequently, we evaluated different ratios of IFN-γ to TNF-α (1:1, 2:1, 1:2), using concentrations of 10 or 20 ng/mL for each cytokine. Within the most effective ratio, cytokine concentrations ranging from 5 to 100 ng/mL were tested. After licensing, the medium was replaced with unsupplemented Human Mesenchymal-LS Basal Medium (Merck, SCMM-BM) for secretome production. To further refine the conditions, we examined the impact of cellular confluence by seeding cells at densities between 60% and 90%, and studied how confluence influences CM production. Finally, we collected CM after 24, 48, or 72 h of incubation to identify the optimal production time. CM was centrifuged at 460 g for 10 min to remove cellular debris, and stored at -80 °C until further analysis.

#### Unlicensed CM

To obtain unlicensed CM, hTERT-AT-MSCs were cultured in complete media until reaching 60–90% confluence. Subsequently, the complete media was replaced with production media, consisting of non-supplemented human Mesenchymal-LS Basal Media (Merck, SCMM-BM). Unlicensed CM was then collected after 24, 48, or 72 h of incubation. The CM was centrifuged at 460 g for 10 min to remove cellular debris before being stored at -80 °C for subsequent analysis.

### Viability and metabolic activity assays

Cell viability was assessed using the LIVE/DEAD^®^ viability/cytotoxicity assay (calcein AM/ethidium homodimer staining, code L3224, Fisher Scientific, Spain). hTERT-AT-MSCs were stained according to the manufacturer’s instructions. After a 30-minute incubation with the dye, fluorescence micrographs were captured using an epi-fluorescence microscope (Nikon TSM). This assay provided a direct measurement of live versus dead cells, allowing for an assessment of cell viability. Metabolic activity was evaluated using the CCK-8 (Sigma Aldrich, Cat. No: 96992), a colorimetric assay based on dehydrogenase activity in viable cells. Briefly, 100 µL of cell suspension was seeded into a 96-well plate, and 10 µL of CCK-8 reagent was added to each well. After a 4 h incubation at 37 °C, absorbance was measured at 450 nm using an Infinite M200 TECAN plate reader.

### Cell doubling time

Cell doubling time was determined by seeding hTERT-AT-MSCs at densities between 2 × 10^4^ and 40 × 10^4^ cells per well in 24-well plates. Cells were detached using trypsinization, stained with Trypan Blue (Fisher Scientific, Cat. No: 15250061), and counted using a TC20 Automated Cell Counter (Bio-Rad) at days 2, 4, and 7. Viable cell counts were used to calculate doubling time.

### Lactate dehydrogenase (LDH) release

LDH release, an indicator of cytotoxicity, was assessed using the LDH cytotoxicity detection kit (Sigma Aldrich, Cat. No: 11644793001). The assay was performed on CM collected at 24, 48, and 72 h, consistent with the timing of the ELISA assays. Absorbance was recorded at 490 nm, with a reference wavelength of 680 nm, in a 96-well plate format. Positive (lysis buffer) and negative (Phosphate-Buffered Saline, PBS) controls were included to determine maximum and spontaneous LDH release, respectively.

LDH release (%) was calculated using the following formula:

1 $$\:LDH\:release\:\%=\:\frac{(Sx-Nc)}{(Pc-Nc)}\times100$$

Where:

- Sx represents the absorbance value of the sample.
- Nc is the absorbance of the negative control (spontaneous LDH release in PBS).
- Pc is the absorbance of the positive control (maximum LDH release with lysis buffer).

This calculation normalizes the LDH release to the maximum possible release (positive control) and expresses it as a percentage relative to the total cytosolic LDH content.

### Bicinchoninic acid assay

Total protein concentration in the CM collected at 72 h was quantified using the BCA protein assay kit (ThermoFisher, Cat. No: 23225), according to the manufacturer’s protocol. Samples were diluted in the appropriate buffer and incubated at 37 °C for 30 min. Absorbance was measured at 562 nm using the Infinite M200 TECAN plate reader.

### MSCs Immunomodulatory CM characterization

The bioactivity of the licensed CM was characterized by measuring the concentrations of immunomodulatory cytokines and growth factors using enzyme-linked immunosorbent assay (ELISA). Specifically, Gal-9 (ThermoFisher, Cat. No: EH390RB), IL-1Ra (Bio-Techne R&D Systems, Cat. No: DRA00B), HGF (Bio-Techne R&D Systems, Cat. No: DHG00), and VEGF (ThermoFisher, Cat. No: EHVEGF) were quantified using commercially available ELISA kits, following the manufacturers’ instructions.

Additionally, a multiplex ELISA array (RayBio^®^ Label-Based L-Series, RayBiotech, USA) was performed to obtain a broader characterization of the MSC-derived secretome in both the control (non-licensed CM) and licensed (60:60 IFN-γ/TNF-α) groups. This semiquantitative antibody-based array allows for the simultaneous detection of multiple analytes within the same sample, providing a comparative analysis of cytokine and growth factor secretion patterns. The following analytes were assessed: interleukin-1 beta (IL-1β), interleukin-6 (IL-6), interleukin-10 (IL-10), interleukin-1 receptor antagonist (IL-1Ra), leukemia inhibitory factor (LIF), monocyte chemoattractant protein-1 (MCP-1), interleukin-8 (IL-8), regulated upon activation, normal T cell expressed and secreted (RANTES), stromal cell-derived factor-1 (SDF-1), hepatocyte growth factor (HGF), vascular endothelial growth factor (VEGF), transforming growth factor beta (TGF-β), TNF-stimulated gene-6 (TSG-6) and Galectin-1 (Gal-1). The assay was conducted according to the manufacturer’s protocol, where samples were biotin-labeled prior to incubation with a pre-coated glass slide containing capture antibodies. Detection was performed using a fluorescence-based system with streptavidin-Cy3 conjugation, and signals were read using a laser fluorescence scanner. Data were normalized to internal positive controls, and results were expressed as relative fluorescence intensity rather than absolute concentrations.

### Functional evaluation of MSCs CM for Immunomodulation

#### PBMCs proliferation assay

The suppressive effect of licensed and unlicensed CM derived from hTERT-AT-MSCs on activated human PBMCs proliferation was also evaluated using a carboxyfluorescein succinimidyl ester (CFSE) proliferation assay. Briefly, PBMCs were suspended in 1 mL of RPMI 1640 medium supplemented with 1% fetal bovine serum (FBS), 1% penicillin/streptomycin (P/S) and 1% L-glutamine, and then labeled with 5 µM CFSE for 15 min. Following labeling, cells were washed with ice-cold PBS supplemented with 1% bovine serum albumin (BSA), centrifuged and then seeded at a density of 2 × 10^5^ cells per well in 96-well plates. The cells were cultured for 5 days in unlicensed and licensed CM or media supplemented with 2.5 µg/mL Concanavalin A and 1.5% FBS. At day 3, half of the media was replaced with fresh unlicensed or licensed CM or media containing 1.5% FBS. On day 5, cells were harvested, and CFSE intensity was measured using flow cytometry. Data acquisition was performed with a MACSQuant^®^ analyzer, and subsequent analysis was conducted using FlowJoTM v10.6.2 Software.

#### Induction of regulatory T cells

To induce Tregs in vitro, CD4 + T lymphocytes were cultured in 24-well plates with RPMI 1640 medium supplemented with 1% FBS, 1% P/S and 1% L-glutamine, at an initial seeding density of 1 × 10^6^ cells/mL. On day 2, cells were harvested, counted and activated with 0.3 µL of Dynabeads Human T-Activator CD3/CD28 (Gibco, 10310614). The activation process was performed under three conditions: (1) control, where cells were activated with Dynabeads alone, which promoted activation and proliferation of CD4 + T lymphocytes without inducing Treg differentiation; (2) positive control, where cells were activated with Dynabeads plus 5 µL/well of Interleukin-2 (IL-2, 10000U, Gibco, 11147528001), which facilitated activation, proliferation and Treg induction; (3) unlicensed and licensed CM, where cells were activated with Dynabeads in their presence. Following a 3-day incubation, the medium was refreshed on day 5, and treatments were re-administered. On day 7, cells were collected, Dynabeads were removed using a magnetic field and the resulting cell population was stained with anti-CD4 (VioBlue^®^REA, 130-114-534), anti-CD25 (PE^®^REA, 130-113-286) and anti-CD127 (APC^®^REA 130-113-407) antibodies for subsequent flow cytometry analysis. Analysis was performed using a MACSQuant^®^ analyzer (Miltenyi Biotec) and data were processed using FlowJoTM v10.6.2 Software (BD Life Sciences, Ashland, OR, USA). For additional co-culture analysis, PBMCs were seeded in 96-well plates and activated with Dynabeads for 7 days. These PBMCs were co-cultured with hTERT-AT-MSCs at various ratios (1:1, 1:2, 1:5, and 1:10 MSCs: PBMCs), with parallel control groups including Dynabeads alone and Dynabeads plus IL-2. Media was refreshed on day 5, and on day 7, cells were collected, Dynabeads were removed, and the cell populations were stained with anti-CD4, anti-CD25, and anti-CD127 antibodies or isotype controls. Flow cytometry analysis was conducted using a MACSQuant^®^ analyzer (Miltenyi Biotec), and data were processed with FlowJoTM v10.6.2 Software (BD Life Sciences, Ashland, OR, USA).

### Data analysis and statistics

Statistical analysis was performed using SPSS 25.0 (IBM). Normality was assessed for each dataset. If data were normally distributed, comparisons between two independent groups were analyzed using the Student’s T-test. For multiple group comparisons, a one-way ANOVA was conducted, with the Levene test employed to evaluate the homogeneity of variances. If variances were homogeneous, the Bonferroni post-hoc test was applied; otherwise, the Tamhane post-hoc test was used. For data that did not follow a normal distribution, the Mann-Whitney test was utilized to assess differences. Results are presented as mean ± standard deviation. All experiments were performed with a minimum of three independent replicates. A significance level of *P* < 0.05 was considered statistically significant.

## Results and discussion

### MSCs licensing strategy: licensing with a single cytokine or in synergy

We initiated our investigation by evaluating the efficacy of licensing MSCs with either single cytokines (IFN-γ or TNF-α) or a combination at a 1:1 ratio, given their well-documented roles in inducing an immunomodulatory phenotype and enhancing the secretion of immunomodulatory factors [18, 23–31, 42]. The 1:1 ratio was initially chosen based on prior literature, where equal concentrations of IFN-γ and TNF-α have been widely used due to their reported synergistic effects on MSC immunomodulation. This condition served as a starting point for comparison before exploring other cytokine ratios (1:1, 2:1, 1:2) in Sect. MSCs licensing strategy: ratios of IFN-γ and TNF-α (1:1, 1:2, 2:1) to determine the most effective balance between immunomodulatory enhancement and cell viability. For the initial evaluation, concentrations of 20 ng/mL for both cytokines were selected, as they are among the most commonly used in previous studies [18, 23–31, 42]. We assessed the impact of these licensing conditions on cell health parameters, including viability, metabolic activity, doubling time, and LDH release into the conditioned media (CM) after 72 h. Additionally, we evaluated the secretion profile (total protein, cytokines, and growth factors) at 24, 48, and 72 h to capture temporal dynamics and their influence on the immunomodulatory and regenerative functions of MSCs. This comprehensive analysis allowed us to compare the effects of licensing with individual cytokines versus their combination on MSC performance.

**Fig. 1:**
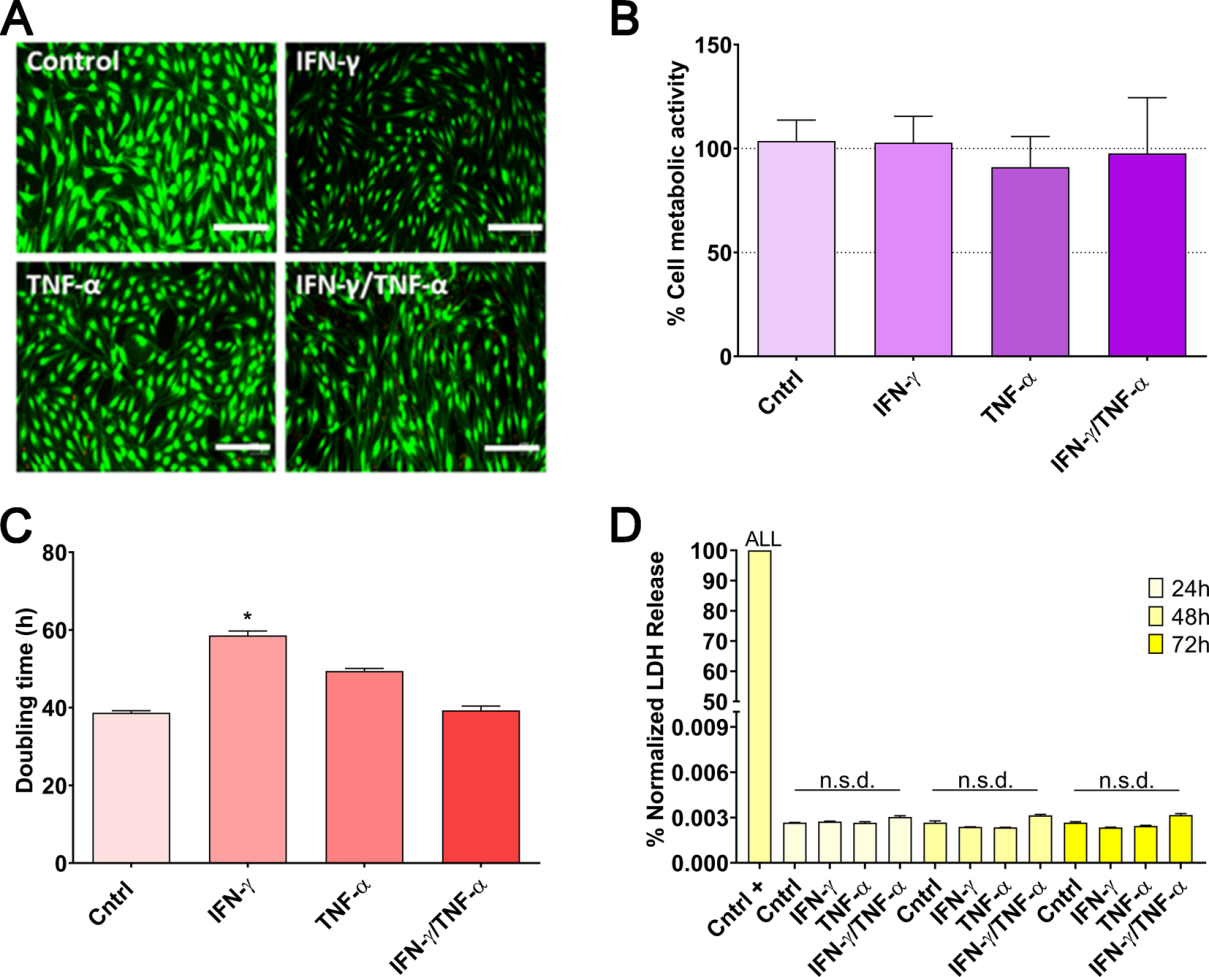
MSC licensing strategy: cell health parameters following stimulation with 20 ng/mL of IFN-γ, TNF-α, or their combination at a 1:1 ratio. (**A**) LIVE/DEAD staining at 72 h. (**B**) Cell metabolic activity at 72 h. (**C**) Doubling time at 72 h. (**D**) LDH release at 24, 48, and 72 h. Data are presented as mean ± SD (*N* = 3 independent experiments, with 5 replicates per group). Statistical significance: *p < 0.05 compared to the unlicensed control group; n.s.d. (non-significant difference) between groups. For LDH release (**D**), the positive control showed statistical significance (*p* < 0.001) for all groups. Statistical analysis was performed using one-way ANOVA followed by Bonferroni post-hoc test for **B**–**D**. Abbreviations: *MSCs: mesenchymal stromal cells. IFN-γ: interferon γ. TNF-α: tumor necrosis factor α. LDH: lactate dehydrogenase*

First, licensing MSCs with IFN-γ or TNF-α at a concentration of 20 ng/mL for 72 h did not affect viability and metabolic activity, as evidenced by the absence of statistically significant differences compared to the unlicensed control group in LIVE/DEAD staining and cell metabolism assays (Fig. 1A-B). However, upon examination of cell doubling time, only the group licensed with IFN-γ exhibited a significantly prolonged doubling time (*p* < 0.05), indicative of impaired cell proliferation, which is a non-desired outcome (Fig. 1C). To ensure that changes in the secretome were not associated by cellular stress or damage, we analyzed LDH content in the CM. LDH release, an indicator of potential cytotoxicity, showed no statistically significant differences between licensed and unlicensed groups at 24, 48, and 72 h (Fig. 1D). This confirms that the observed changes in the secretome are not attributable to cellular damage. It is important to note that excessive LDH release may result in the release of damage-associated molecular patterns (DAMPs), which could compromise the therapeutic efficacy of the CM by triggering unwanted immune responses [43].

**Fig. 2:**
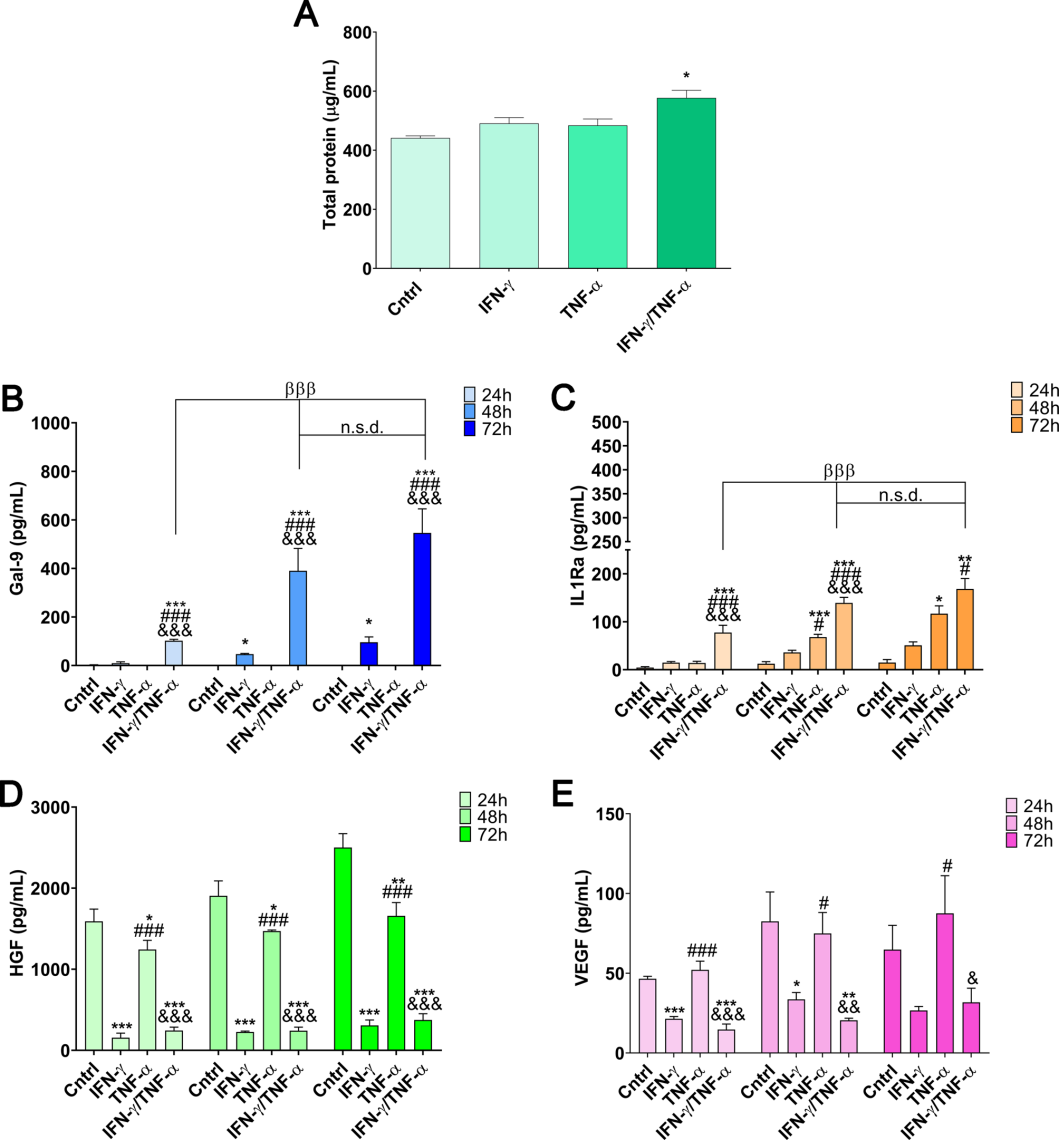
Secretory profile of MSCs licensed with 20 ng/mL of IFN-γ, TNF-α, or their combination (1:1 ratio) at different time points. (**A**) Total protein in the secretome at 72 h. (**B**–**E**) Gal-9, IL-1Ra, HGF, and VEGF levels in the secretome at 24, 48, and 72 h. Data are presented as mean ± SD (*N* = 3 independent experiments, with 5 replicates per group). Statistical significance: *p < 0.05, **p < 0.01, ***p < 0.001 compared to the unlicensed control group; ^#^*p* < 0.05, ^##^*p* < 0.01, ^###^*p* < 0.001 compared to the IFN-γ licensed group; ^&^*p* < 0.05, ^&&^*p* < 0.01, ^&&&^*p* < 0.001 compared to the TNF-α licensed group; ^βββ^*p* < 0.001 compared against the 24-hour IFN-γ/TNF-α licensed group (1:1 ratio); n.s.d. (non-significant difference) compared against the 48-hour IFN-γ/TNF-α licensed group (1:1 ratio). Statistical analysis was performed using one-way ANOVA with the Bonferroni post-hoc test in **A**–**E**. Abbreviations: *MSCs: mesenchymal stromal cells. IFN-γ: interferon γ. TNF-α: tumor necrosis factor α. Gal-9: galectin-9. IL-1Ra: interleukin-1 receptor antagonist. HGF: hepatocyte growth factor. VEGF: vascular endothelial growth factor*

As for the secretion profile, the analysis of total protein content at 72 h did not reveal statistically significant differences compared to the unlicensed control group, except for the IFN-γ/TNF-α licensed group, which demonstrated a higher protein content (*p* < 0.05) (Fig. 2A). This time point was chosen based on the assumption that prolonged incubation reflects a steady-state of secretion.

In contrast, the secretion of specific immunomodulatory factors was evaluated at 24, 48, and 72 h to capture their temporal dynamics and identify potential peaks in factor release. The analysis included the immunomodulatory cytokines Gal-9 and IL-1Ra, as well as the growth factors HGF and VEGF. Licensing with the combination of IFN-γ and TNF-α significantly increased the concentrations of Gal-9 and IL-1Ra, as compared to licensing protocols with single cytokines. Given their essential role in MSC-mediated immunomodulation, these markers were chosen for further evaluation. Gal-9 is a potent immunosuppressive molecule that interacts with the Tim-3 receptor on T cells, leading to the inhibition of effector T cell activity and promoting Tregs differentiation [44]. Additionally, it modulates cytokine secretion by various immune cell populations, contributing to immune homeostasis. Similarly, IL-1Ra functions as an anti-inflammatory mediator by competitively inhibiting IL-1β signaling, preventing the activation of pro-inflammatory cascades and reducing immune cell recruitment and activation [45]. Their increased presence in the licensed secretome highlights the immunosuppressive shift induced by IFN-γ/TNF-α priming. The optimal production time was achieved after 48 h, as observed by the absence of statistically significant differences between 48 and 72 h (Fig. 2B-C).

However, concerning growth factors, licensing with IFN-γ negatively influenced their secretion, resulting in lower concentrations compared to the control group. Conversely, TNF-α did not affect secretion relative to the control. Consequently, groups licensed with both IFN-γ and TNF-α also exhibited reduced concentrations of growth factors (Fig. 2D-E). To the best of our knowledge, while the potential for synergistic effects of IFN-γ and TNF-α on MSC licensing has been previously suggested, this study provides the first detailed evidence demonstrating that their combination not only enhances the secretion of immunomodulatory factors but also simultaneously reduces growth factor levels in the MSC secretome.

Given that the goal is to produce a secretome enriched with immunomodulatory factors for treating IMIDs, the most effective strategy is licensing cells with both cytokines. These findings emphasize the critical role of using a cocktail of IFN-γ and TNF-α, particularly in enhancing immunomodulatory factors such as Gal-9 or IL1-Ra. This increase appears to be synergistic rather than merely additive, underlining the importance of cytokine combination strategies for optimizing MSC secretome production.

### MSCs licensing strategy: ratios of IFN-γ and TNF-α (1:1, 1:2, 2:1)

Given that IFN-γ and TNF-α were previously selected based on their well-documented roles in MSC licensing and their widespread use in the literature [18, 23–25, 27–30, 33–36], we next sought to determine the optimal ratio between these cytokines to maximize their synergistic effects on CM composition and immunomodulatory potential. To address this, we utilized the most commonly reported cytokine concentrations in the literature, namely 20 ng/mL and 10 ng/mL for IFN-γ and TNF-α [18, 23–31, 42]. Licensing was performed at these concentrations, establishing the ratios of 1:1 (20/20 ng/mL IFN-γ/TNF-α), 2:1 (20/10 ng/mL IFN-γ/TNF-α) and 1:2 (10/20 ng/mL IFN-γ/TNF-α), with unlicensed cells serving as the control group. Subsequently, experiments were replicated as described in MSCs licensing strategy: licensing with a single cytokine or in synergy.

**Fig. 3:**
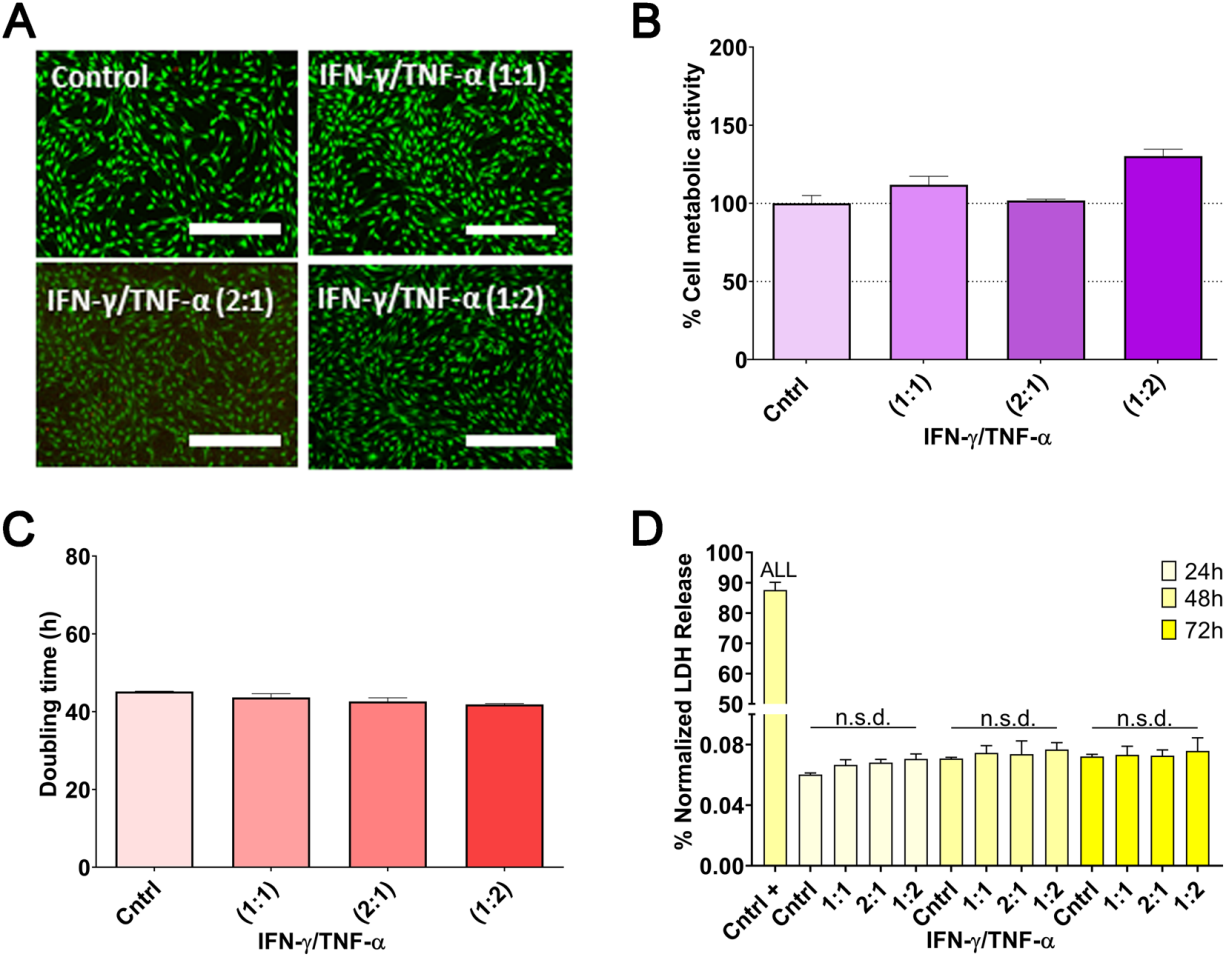
Cell health parameters of MSCs licensed at different cytokine ratios of IFN-γ and TNF-α (1:1, 1:2, 2:1). (**A**) LIVE/DEAD staining at 72 h. (**B**) Cell metabolic activity at 72 h. (**C**) Doubling time at 72 h. (**D**) LDH release at 24, 48, and 72 h. Data are presented as mean ± SD (*N* = 3 independent experiments, with 4 replicates per group). Statistical significance: n.s.d. (non-significant difference) between groups. For LDH release (**D**), the positive control showed statistical significance (*p* < 0.001) for all groups. Statistical analysis was performed using one-way ANOVA followed by the Bonferroni post-hoc test in **B**–**D**. Abbreviations: *MSCs: mesenchymal stromal cells. IFN-γ: interferon γ. TNF-α: tumor necrosis factor α. LDH: lactate dehydrogenase*

In terms of cell viability, both LIVE/DEAD staining and metabolic activity experiments revealed no statistically significant differences among the tested ratios, as evidenced by micrographs indicating consistent viability under all conditions (Fig. 3A-B). Additionally, no significant differences were observed in doubling time and LDH release experiments (Fig. 3C-D), indicating that none of the tested ratios negatively affected cellular health.

**Fig. 4:**
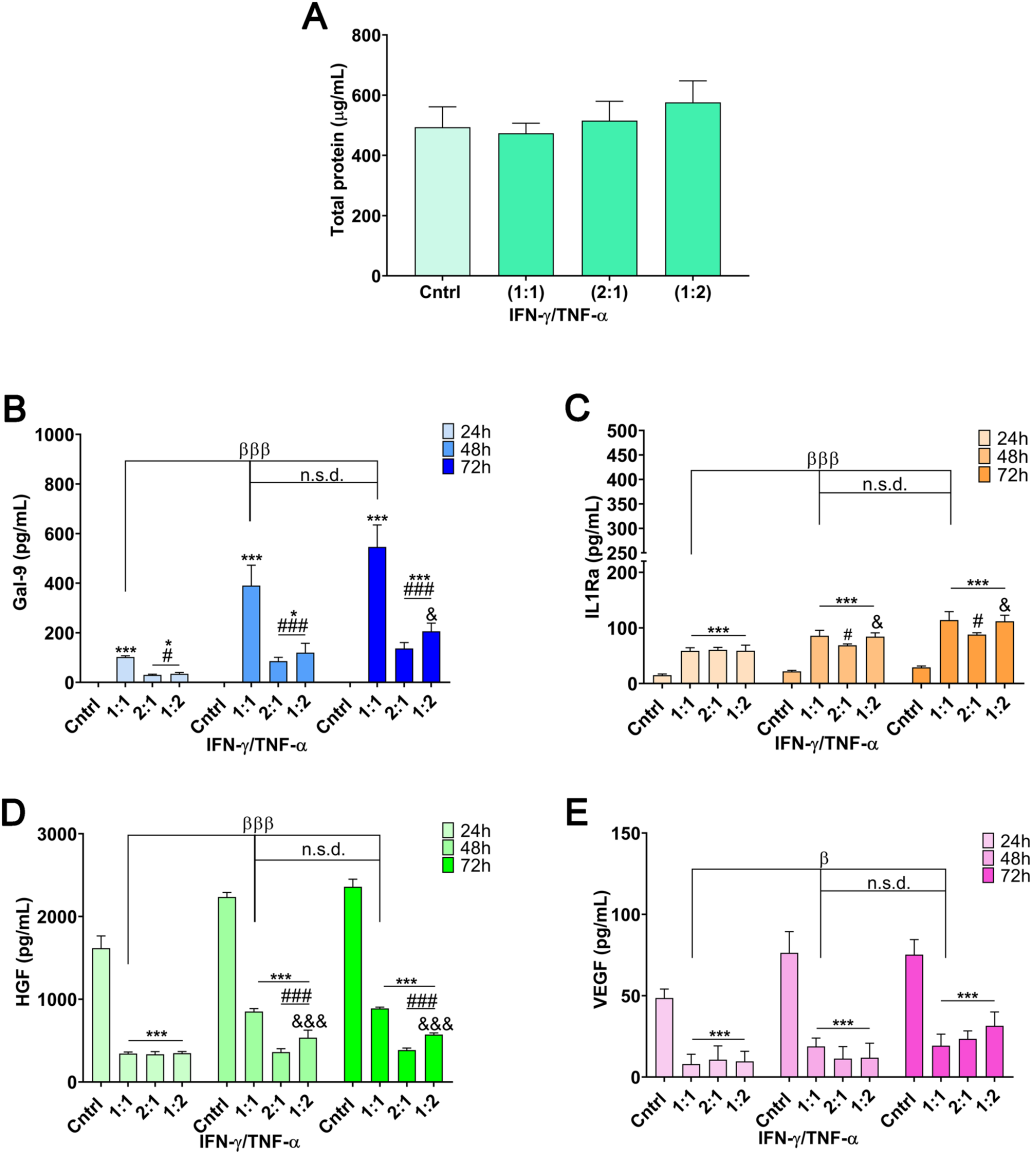
MSCs licensing strategy: effects on the secretory profile at different time points and ratios of IFN-γ and TNF-α (1:1, 1:2, and 2:1). (**A**) Total protein in the secretome at 72 h. (**B**-**E**) Gal-9, IL1-Ra, HGF and VEGF in the secretome at 24, 48 and 72 h. Data are presented as mean ± SD. (*N* = 3 independent experiments, with 4 replicates per group). Statistical significance: *p < 0.05 and ***p < 0.001 compared to the unlicensed control group. ^#^*p* < 0.05 and ^###^*p* < 0.001 compared to the 1:1 IFN-γ/TNF-α licensed group. ^&^*p* < 0.05 and ^&&&^*p* < 0.001 compared to the 2:1 IFN-γ/TNF-α licensed group. ^β^*p* < 0.05 and ^βββ^*p* < 0.001 compared between 24 h 1:1 IFN-γ/TNF-α licensed group and n.s.d. (non-significant difference) compared between 48 h and 72 h 1:1 IFN-γ/TNF-α licensed group. Statistical analysis was performed using One-way ANOVA with the Bonferroni post-hoc test in **A**-**E**. Abbreviations: *MSCs: mesenchymal stromal cells. IFN-γ: interferon γ. TNF-α: tumor necrosis factor α. Gal-9: galectin 9. IL-1Ra: interleukin-1 receptor antagonist. HGF: hepatocyte growth factor. VEGF: vascular endothelial growth factor*

In the context of the secretion profile, no significant differences in total protein levels were observed between the tested ratios (Fig. 4A). However, when examining the secretion of immunomodulatory cytokines, the 1:1 licensing ratio yielded the highest concentration of Gal-9, highlighting its superior effectiveness in enhancing this key factor (Fig. 4B). Notably, the CM collected at 48 h exhibited optimal concentrations of Gal-9, demonstrating a significant increase compared to the 24 h CM (*p* < 0.001), with no statistically significant differences observed between the 48 and 72 h CM (Fig. 4B). Similarly, for IL-1Ra, the 48 h CM yielded optimal concentrations, with the 1:1 and 1:2 ratios showing the highest concentrations, and no significant differences between them (Fig. 4C).

Regarding growth factors, and consistent with previous observations, their secretion was markedly reduced in all groups tested under licensing protocols. However, from 48 h onwards, the 1:1 ratio showed higher HGF levels compared to the 2:1 and 1:2 ratios, despite the overall decrease. This time point was identified as the optimal one, with no significant improvements observed at 72 h (Fig. 4D). Similarly, VEGF levels followed a comparable trend across all tested ratios, with optimal outcomes also observed at 48 h (Fig. 4E). These findings align with previous reports indicating that IFN-γ and TNF-α licensing may lead to a shift in MSC function, prioritizing immunomodulatory effects over regenerative potential [23–30]. While HGF and VEGF are key mediators of tissue repair and angiogenesis, their reduction following pro-inflammatory licensing suggests a functional adaptation toward immune regulation. However, this does not necessarily compromise tissue repair, as the immunosuppressive effects of the licensed secretome can contribute to a pro-repair microenvironment by dampening excessive inflammation and promoting immune homeostasis. This observation highlights the ability to fine-tune the MSC secretome depending on the desired therapeutic outcome. For applications requiring enhanced regenerative properties, a non-licensed secretome could be a more suitable approach. However, for therapeutic strategies prioritizing immunomodulation, licensing with IFN-γ and TNF-α remains the optimal choice.

Consequently, based on these results, the IFN-γ/TNF-α 1:1 ratio emerged as the most effective approach for achieving an optimal secretory profile of immunomodulatory factors, particularly at the 48 h time point. This ratio demonstrated a significant enhancement in the secretion of key cytokines such as Gal-9 and IL-1Ra, while maintaining favorable cell viability and metabolic activity. Building on this foundation, subsequent experiments were designed to explore additional variables under the 1:1 ratio, progressively refining the framework of the licensing strategy.

### MSCs licensing in 1:1 sinergy ratio with IFN-γ and TNF-α: concentration and time optimization

In our pursuit to optimize the immunomodulatory secretome of MSCs, we meticulously explored the impact of varying cytokine concentrations within the 1:1 synergy ratio (5–100 ng/mL) on both cellular health and CM composition. Cell viability (LIVE/DEAD staining), metabolic activity (CCK assay), and proliferation (doubling time) were assessed at 72 h, while LDH release was analyzed at multiple time points (24, 48, and 72 h).

**Fig. 5:**
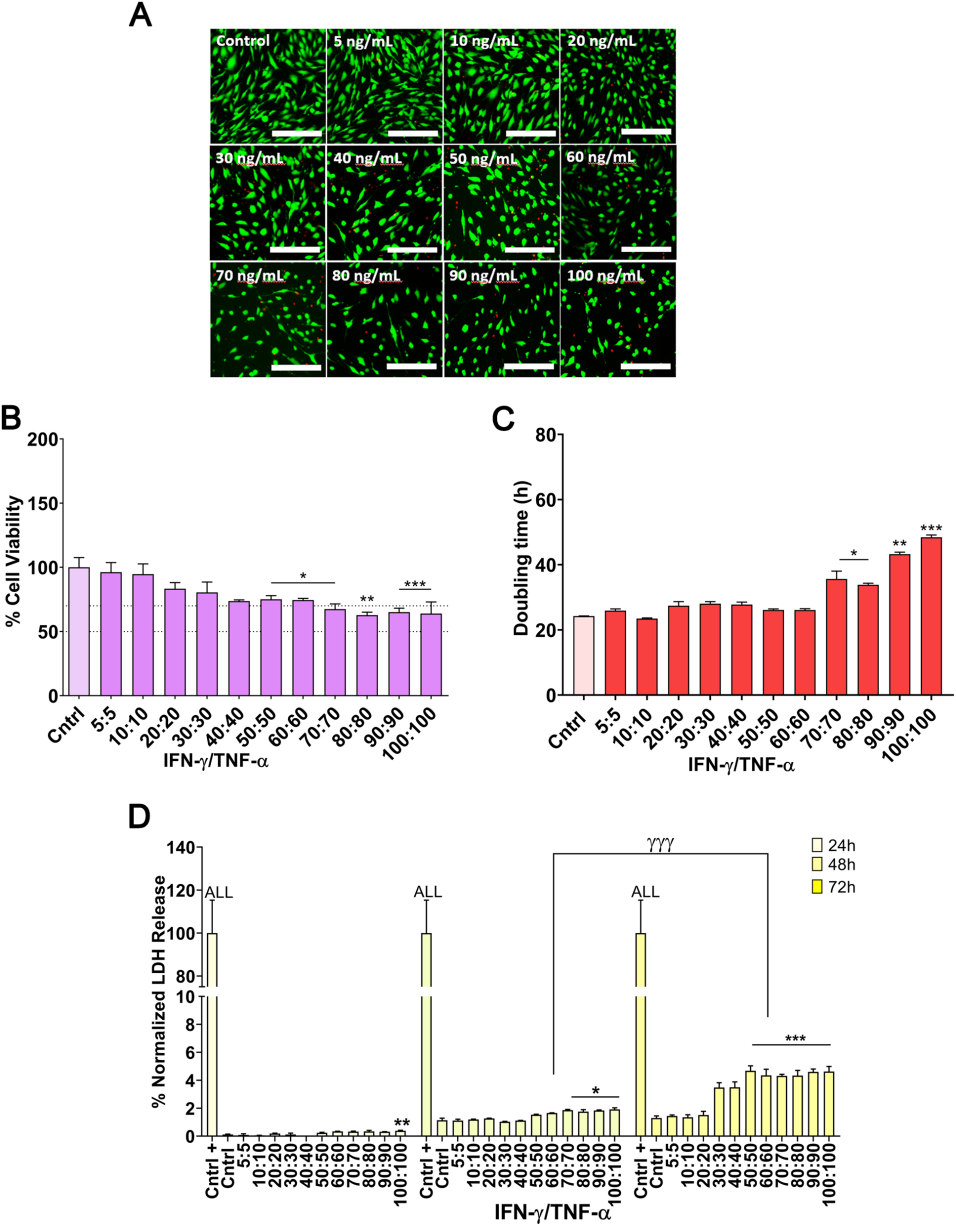
Optimization of MSC licensing: impact of cytokine concentration and incubation time on cell health at a 1:1 ratio of IFN-γ and TNF-α. (**A**) LIVE/DEAD staining at 72 h. (**B**) Cell metabolic activity at 72 h. (**C**) Doubling time at 72 h. (**D**) LDH release at 24, 48, and 72 h. All parameters were evaluated across cytokine concentrations ranging from 5 to 100 ng/mL (IFN-γ and TNF-α, 1:1 ratio). Data are presented as mean ± SD (*N* = 3 independent experiments, with 4 replicates per group). Statistical significance: *p < 0.05, **p < 0.01, ***p < 0.001 compared to the unlicensed control group; ^γγγ^*p* < 0.001 and compared to the 60/60 ng/mL IFN-γ/TNF-α licensed group between 48 and 72 h of CM production. For LDH release (**D**), the positive control showed statistical significance (*p* < 0.001) for all groups. Statistical analysis was performed using one-way ANOVA followed by Bonferroni post-hoc test in **B**–**D**. Abbreviations: *MSCs: mesenchymal stromal cells. IFN-γ: interferon γ. TNF-α: tumor necrosis factor α. CM: conditioned media. LDH: lactate dehydrogenase*

Cell viability assays, as depicted in LIVE/DEAD staining micrographs and confirmed through CCK assays, revealed significant patterns. Licensing with cytokines at 40 ng/mL led to a decrease in cellular viability, although viability remained around 70% up to 60 ng/mL, which is generally considered an acceptable threshold for viability assays. Beyond this concentration, viability declined further (*p* < 0.01 and *p* < 0.001) (Fig. 5A-B). Consistent with this, doubling time analysis showed no significant changes in cell proliferation up to 60 ng/mL, but a statistically significant increase in doubling time was observed beyond 70 ng/mL (Fig. 5C).

Regarding LDH secretion, distinct patterns emerged across the tested concentrations and collection times. At 24 h, only the 100 ng/mL concentration showed a statistically significant increase in LDH release compared to the unlicensed control group (*p* < 0.01). At 48 h, statistically significant differences were observed starting at 70 ng/mL (*p* < 0.05), while at 72 h, differences became apparent from 50 ng/mL (*p* < 0.001). Notably, when comparing CM collected at 48 and 72 h for the 60 ng/mL concentration, a significant difference in LDH release was detected (*p* < 0.001). These results suggest that a concentration of 60 ng/mL with a collection time of 48 h represents the optimal balance for effective cytokine licensing, as higher concentrations or longer collection times result in increased cytotoxicity.

Additionally, Supplementary Fig. 1 shows micrographs captured at 0, 24, 48, and 72 h, illustrating MSC morphology under different licensing conditions. The images were obtained using an automated imaging system, and the groups examined include MSCs without licensing (0 ng/mL), as well as MSCs licensed with 20 ng/mL, 60 ng/mL, and 100 ng/mL of IFN-γ and TNF-α. These images further highlight that, although MSC morphology appears generally preserved following licensing, subtle changes can be observed at higher cytokine concentration 100 ng/mL, particularly at later time points (48 and 72 h). These morphological observations align with the previously discussed health parameters, indicating that 60 ng/mL serves as the threshold concentration below which cytotoxic effects are not observed, with mild cytotoxic effects appearing at higher concentrations. Thus, while the overall cellular integrity is maintained, the findings reinforce the need for careful selection of licensing conditions to balance morphological preservation with optimal immunomodulatory effects.

**Fig. 6:**
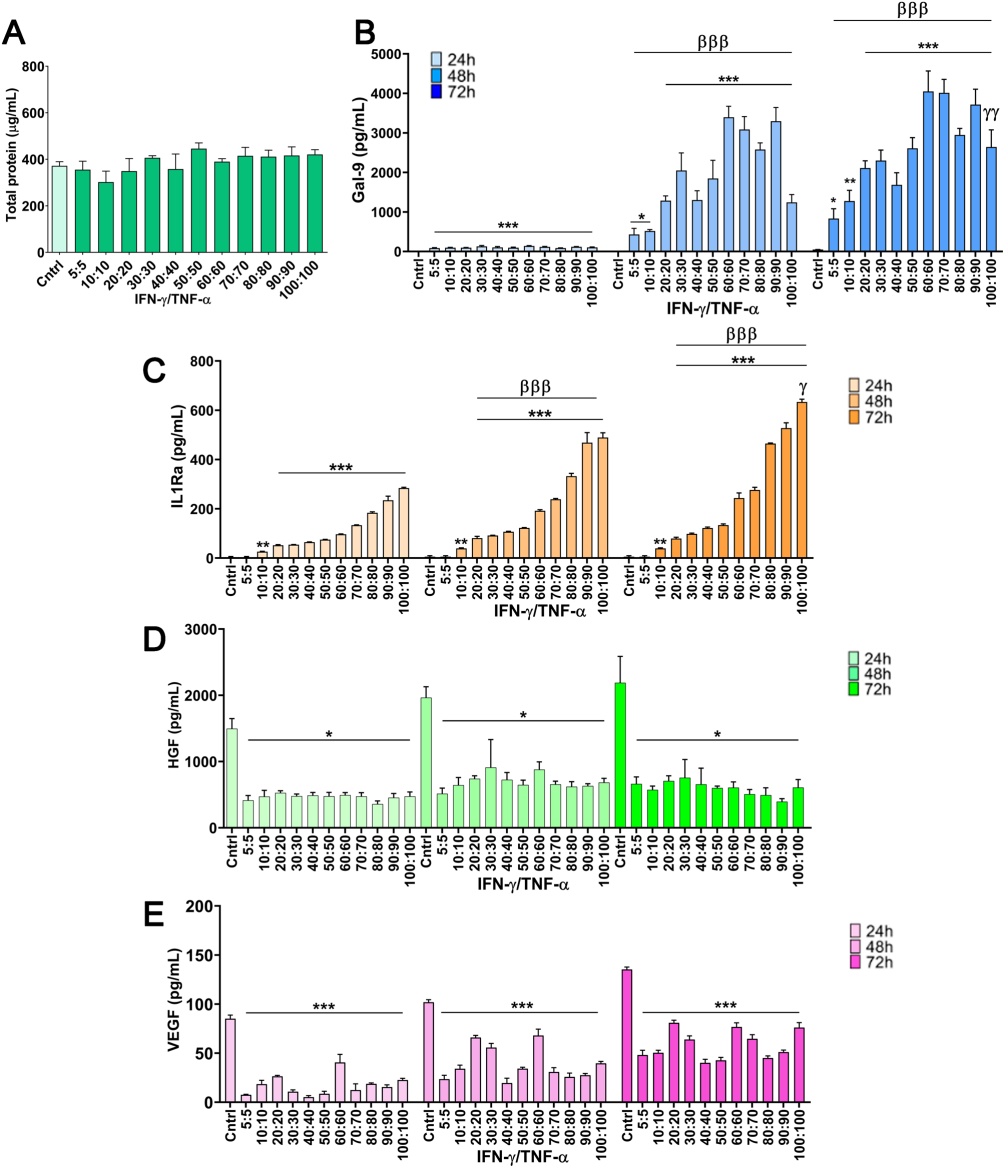
Analysis of bioactive factors in the conditioned media (CM) across different cytokine concentrations (5–100 ng/mL) and time points (24, 48, and 72 h) using IFN-γ and TNF-α at a 1:1 ratio. (**A**) Total protein in the secretome at 72 h. (**B**-**E**) Gal-9, IL1-Ra, HGF and VEGF levels at 24, 48, and 72 h. Data are presented as mean ± SD. (*N* = 4 independent experiments, with 4 replicates per group). Statistical significance: *p < 0.05, **p < 0.01 and ***p < 0.001 compared to the unlicensed control group. ^βββ^*p* < 0.001 compared between 24 h licensed group. ^γ^*p* < 0.05 between 48 h licensed group. (One-way ANOVA with the Bonferroni post-hoc test in **A**-**E**. Abbreviations: *IFN-γ: interferon γ. TNF-α: tumor necrosis factor α. Gal-9: galectin 9. IL-1Ra: interleukin-1 receptor antagonist. HGF: hepatocyte growth factor. VEGF: vascular endothelial growth factor*

Having established the optimal cytokine concentration for preserving cell health, we next investigated its impact on the MSC secretory profile. Total protein content analysis did not yield statistically significant differences among groups, indicating a consistent protein production profile across varying cytokine concentrations (Fig. 6A). Further analysis of bioactive factors revealed key insights into the effects of different cytokine concentrations. For Gal-9, licensing with 60 ng/mL cytokines led to the highest levels in the CM, with no significant differences observed between the 48 h and 72 h time points (Fig. 6B), confirming 60 ng/mL as the optimal concentration for maximizing Gal-9 secretion. Interestingly, the secretion pattern of Gal-9 exhibited a non-linear response across different cytokine concentrations, showing an initial decrease followed by a subsequent increase at higher concentrations. This biphasic trend may reflect an adaptive regulatory mechanism within MSCs, where early stress responses to IFN-γ/TNF-α licensing transiently modulate the expression of certain immunomodulatory molecules. Previous studies suggest that MSCs dynamically regulate their galectin-9 secretion through NF-κB and STAT-dependent pathways, which may become more activated as cytokine stimulation intensifies [46]. The increase observed at higher concentrations may thus indicate an enhanced activation state, reinforcing the immunomodulatory function of the secretome at optimal licensing conditions. Conversely, IL-1Ra levels exhibited a more dynamic response across concentrations and time points. Licensing from 20 ng/mL to 100 ng/mL resulted in significant differences in IL-1Ra secretion when comparing CM collected at 24 and 48 h (*p* < 0.001). When comparing 48 and 72 h, significant differences were observed only at the 100 ng/mL concentration (*p* < 0.05) (Fig. 6C).

In contrast, growth factor levels (HGF and VEGF) remained significantly lower across all cytokine concentrations compared to unlicensed controls (Fig. 6D-E). This reinforces previous findings indicating a shift in MSC function from a regenerative to an immunomodulatory profile upon licensing. This finding supports the selection of 60 ng/mL as the optimal concentration, as it allows for the highest secretion of anti-inflammatory cytokines, such as IL-1Ra and Gal-9, while maintaining acceptable levels of cellular health.

### MSCs secretome optimization in terms of confluence

To further refine the MSCs secretome for enhanced immunomodulatory potential, we investigated the effect of cellular confluence on secretome composition. This study builds upon the previously established assays that evaluate cellular viability, total protein content, growth behavior, LDH release, and the secretion of bioactive factors such as Gal-9, IL1-Ra, HGF, and VEGF. Various levels of cellular confluence ranging from 60 to 90% were systematically analyzed to discern their impact on the resultant CM.

**Fig. 7:**
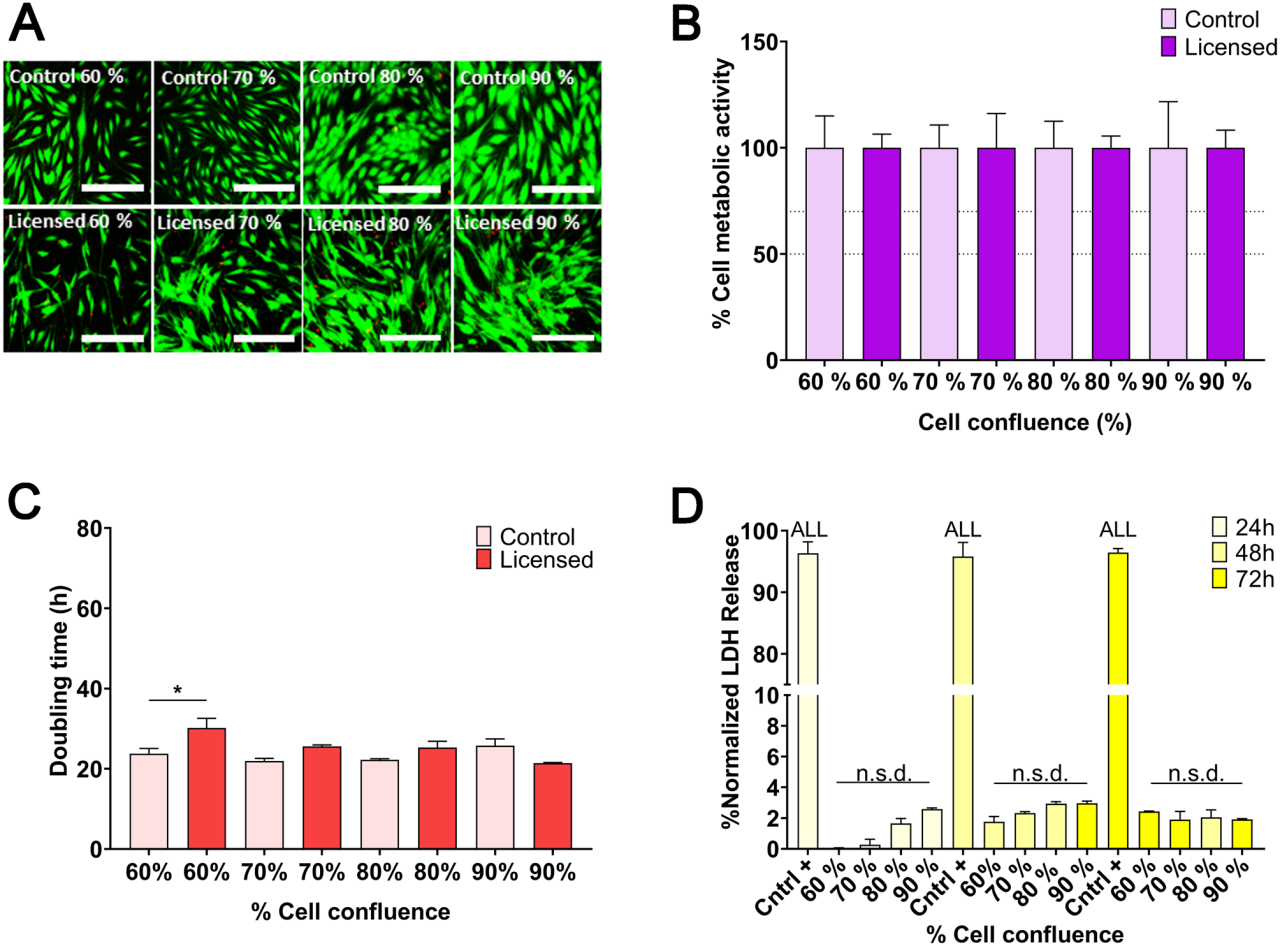
Optimization of MSC secretome production: influence of seeding confluence (60–90%) on cell health after licensing with 20 ng/mL IFN-γ and TNF-α (1:1 ratio). (**A**) LIVE/DEAD staining at 72 h. (**B**) Cell metabolic activity at 72 h. (**C**) Doubling time at 72 h. (**D**) LDH release at 24, 48, and 72 h across different confluence levels. Data are presented as mean ± SD (*N* = 4 independent experiments, with 4 replicates per group). Statistical significance: *p < 0.05 and n.s.d. (non-significant difference) between groups. For LDH release (**D**), the positive control showed statistical significance (*p* < 0.001) for all groups. Statistical analysis was performed using one-way ANOVA followed by Bonferroni post-hoc test in **B**–**D**. Abbreviations: MSCs: mesenchymal stromal cells. IFN-γ: interferon γ. TNF-α: tumor necrosis factor α. LDH: lactate dehydrogenase

Initially, cellular viability was assessed through LIVE/DEAD staining and cell metabolism analysis, comparing cells cultured at different confluences with and without licensing with pro-inflammatory cytokines at a concentration of 20 ng/mL. This concentration was selected based on its widespread use in the literature, where it has been shown to effectively induce an immunomodulatory phenotype in MSCs without compromising cellular viability or function [18, 23, 25–27]. No detrimental effects on cellular viability were observed across different confluences (Fig. 7A-B). Growth kinetics analysis indicated no statistically significant differences in cell doubling rates between control and treated groups, suggesting stable growth profiles regardless of confluence levels (Fig. 7C). To further evaluate cellular integrity, LDH release assays were conducted at different time points (24, 48, and 72 h) following licensing. No significant increase in LDH release was observed in either the unlicensed CM or the licensed CM, indicating that the secretome production process did not induce cytotoxic effects, regardless of cell confluence (Fig. 7D).

**Fig. 8:**
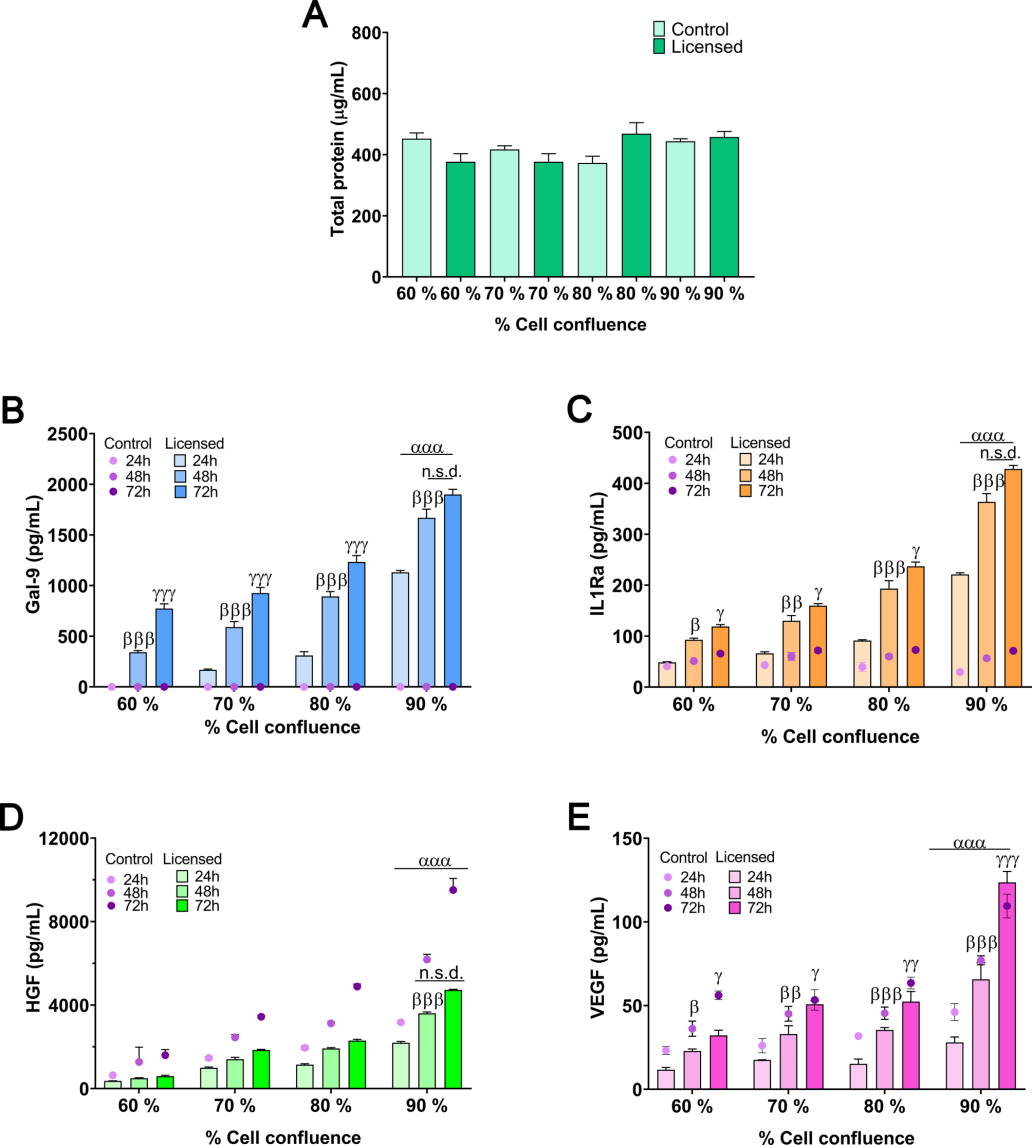
MSC secretory profile at different seeding confluences (60–90%) after licensing with 20 ng/mL IFN-γ and TNF-α at a 1:1 ratio. (**A**) Total protein content in the secretome at 72 h. (**B**-**E**) Gal-9, IL-1Ra, HGF, and VEGF concentrations in the secretome at 24, 48, and 72 h across different confluence groups. Data are presented as mean ± SD. (*N* = 4 independent experiments, with 4 replicates per group). Statistical significance: ^β^*p* < 0.05, ^ββ^*p* < 0.01, ^βββ^*p* < 0.001 compared between 24 h licensed group. ^γ^*p* < 0.05, ^γγ^*p* < 0.01, ^γγγ^*p* < 0.001 and n.s.d. (non-significant difference) between 48 h licensed groups. ^ααα^*p* < 0.001 comparing different confluence levels (60%, 70%, 80%, and 90%). (One-way ANOVA with the Bonferroni post-hoc test in B-I). Abbreviations: MSCs: mesenchymal stromal cells. IFN-γ: interferon γ. TNF-α: tumor necrosis factor α. Gal-9: galectin-9; IL-1Ra: interleukin-1 receptor antagonist; HGF: hepatocyte growth factor; VEGF: vascular endothelial growth factor

An important observation in this study was the behavior of total protein content across varying confluence levels under licensed conditions at 72 h (Fig. 8A). Typically, higher cellular confluence is expected to yield greater total protein secretion due to the increased number of cells in culture. However, our results showed that total protein content remained stable across all confluence levels in the unlicensed controls. In contrast, in the licensed groups, total protein content appeared slightly higher at higher confluence levels, although these differences were not statistically significant. This result is particularly interesting, as one would expect total protein secretion to increase with confluence. A plausible explanation is that, at 72 h, all confluence levels may have reached a plateau in cell growth, possibly due to space limitations for adhesion or contact inhibition. This plateau effect could lead to a similar cumulative protein secretion across conditions, regardless of initial confluence.

This stability in protein production under licensing ensures that comparisons of cytokine and growth factor levels are valid, isolating the effects of confluence on secretome quality rather than quantity. Regarding the secretion of bioactive factors, an upward trend in the levels of Gal-9, IL1-Ra, HGF, and VEGF was observed with increasing cellular confluence (Fig. 8B-E). However, a closer analysis reveals that the secretion rate remains relatively constant during the first 48 h, after which it begins to decline. This decline in production rate leads to a loss of proportionality between time points, with the differences between 72 and 48 h showing non-significant differences compared to the more pronounced differences observed between 48 and 24 h. This effect becomes more evident at higher cellular confluence levels, where the reduced secretion rate beyond 48 h results in non-significant differences when comparing 48 and 72 h.

Interestingly, although total protein levels remain comparable between licensed and unlicensed cells, licensing induces substantial alterations in the relative abundance of key immunomodulatory and regenerative factors. These significant changes in secretome composition, rather than variations in overall protein content, suggest that MSCs undergo an adaptive reprogramming of their secretory machinery in response to external stimuli.

This phenomenon can be attributed to two main factors. First, the increase in cell number over time, which is more evident at lower confluences where cell proliferation is still active, whereas at higher confluence levels, cells are closer to saturation. Second, the cumulative effect of CM production time, as the impact of cytokine stimulation is transient and gradually diminishes. This transient effect has been previously reported, where the overexpression of key immunomodulatory genes, such as IDO and Gal-9, disappeared by 72 h following licensing [47]. These findings suggest that beyond 48 h, the sustained production of secreted factors is not solely dictated by confluence but also by the natural attenuation of the licensing response over time.

The continuous rise in VEGF at 72 h may be linked to microenvironmental changes, such as reduced oxygen availability. However, the secretion peaks of Gal-9, IL-1Ra, and HGF – key factors for immunomodulation and regenerative effects – are observed at 48 h. This time point represents a balance between the cumulative effect of secreted factors and the timeframe during which the overexpression of licensing-responsive genes remains active, reinforcing 48 h as the optimal collection time for applications prioritizing these outcomes.

A confluence of 90% was identified as optimal, maximizing immunomodulatory properties while preserving cell viability. These findings indicate that improvements in bioactivity stem from qualitative changes in CM composition rather than increased cell numbers.

### MSCs secretome characterization and functional studies

Following the identification of optimal licensing conditions based on cellular health and secretory profiles, we characterized the secretion profile of key bioactive factors using a multiplex ELISA. This analysis aimed to provide a comprehensive overview of the MSC-derived secretome under inflammatory licensing prior to functional evaluation.

To facilitate interpretation, the detected factors were grouped into three main functional categories: cytokines (IL-1β, IL-6, IL-10, IL-1Ra, LIF), chemokines (MCP-1, IL-8, RANTES, SDF-1), and growth factors (HGF, VEGF, TGF-β), acknowledging that several molecules – such as TGF-β or HGF – may play dual roles in both regeneration and immune regulation depending on context. In addition, immunomodulatory proteins such as TSG-6 and Galectin-1 were also analyzed given their known roles in immune modulation.

**Fig. 9:**
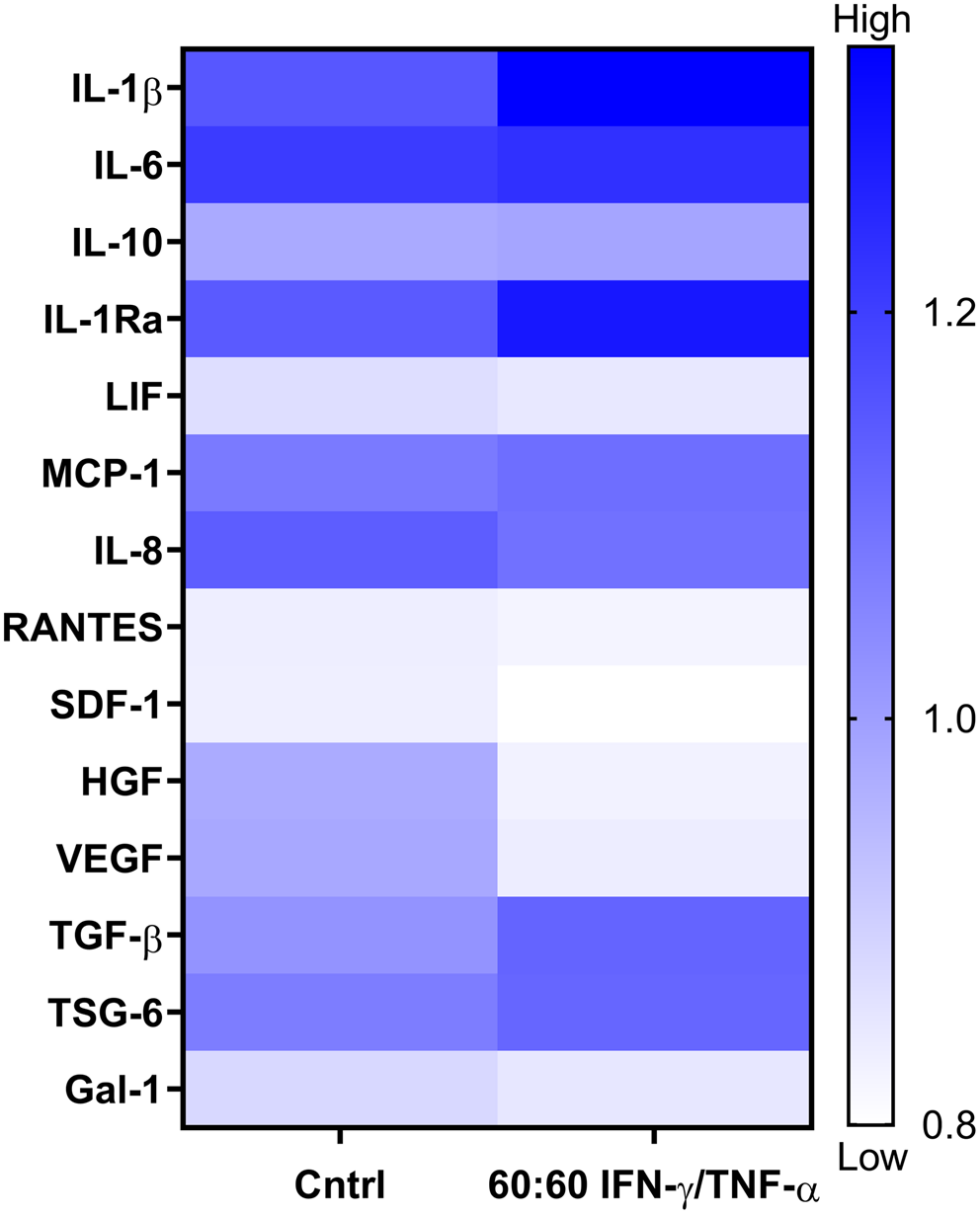
Heatmap showing the secretion levels of cytokines, chemokines, growth factors, and immunomodulatory factors in MSC-conditioned media (CM) after licensing with 60 ng/mL IFN-γ and TNF-α (1:1), assessed by multiplex ELISA. Factors include cytokines (IL-1β, IL-6, IL-10, IL-1Ra, LIF), chemokines (MCP-1, IL-8, RANTES, SDF-1), growth factors (HGF, VEGF, TGF-β), and immunomodulatory proteins (TSG-6, Galectin-1). Data are presented as relative fluorescence intensity (RFI). Abbreviations: *IFN-γ: interferon γ; TNF-α: tumor necrosis factor α; IL: interleukin; LIF: leukemia inhibitory factor; MCP-1: monocyte chemoattractant protein-1; RANTES: regulated upon activation*,* normal T cell expressed and secreted; SDF-1: stromal cell-derived factor-1; HGF: hepatocyte growth factor; VEGF: vascular endothelial growth factor; TGF-β: transforming growth factor-β; TSG-6: TNF-stimulated gene-6; Gal-1: galectin-1*

The heatmap in Fig. 9 illustrates the shift in the MSC secretome following cytokine licensing.

Among pro-inflammatory cytokines, IL-1β was markedly upregulated, IL-6 remained stable, and IL-8 decreased slightly. In terms of anti-inflammatory and immunoregulatory molecules, IL-1Ra and TGF-β exhibited a clear increase upon licensing. Among chemokine regulation, MCP-1 was increased, while RANTES and SDF-1 decreased, reflecting a selective modulation of chemotactic signaling pathways. Regarding growth factors, HGF and VEGF showed a slight decrease in the licensed CM, while TGF-β was upregulated, indicating a functional transition from regenerative to regulatory signaling. Additionally, TSG-6 and Galectin-1 showed visible differences in the heatmap, particularly TSG-6, which increased in response to licensing.

These results indicate that IFN-γ/TNF-α licensing reprograms the MSC secretory profile, shifting the balance between immunomodulatory and regenerative outputs. Specifically, it enhances the secretion of key immune-regulatory factors while differentially modulating inflammatory, chemotactic, and regenerative signals. Figure 9 summarizes these alterations and provides a detailed overview of the bioactive factors modulated by inflammatory licensing.

After establishing the profile of key bioactive factors within the licensed CM, we proceeded to evaluate its immunomodulatory capacity through functional assays, focusing on the inhibition of human PBMC activation and the induction of Tregs.

To this end, three groups of CM were selected for functional studies: (1) CM licensed with 20 ng/mL of IFN-γ/TNF-α, representing the lowest concentration commonly used in the literature, which did not induce any detectable adverse effects on cell health; (2) CM licensed with 60 ng/mL of IFN-γ/TNF-α, identified as the optimal concentration for maximizing the secretion of immunomodulatory factors while preserving cell viability; and (3) CM licensed with 100 ng/mL of IFN-γ/TNF-α, representing the maximum tested concentration, which led to significant cytotoxic effects, highlighting the limits of cellular tolerance and viability under these conditions. These assays were performed using CM produced at 48 h with 90% cellular confluence, as this condition was identified as the most favorable for generating a functionally potent secretome.

**Fig. 10:**
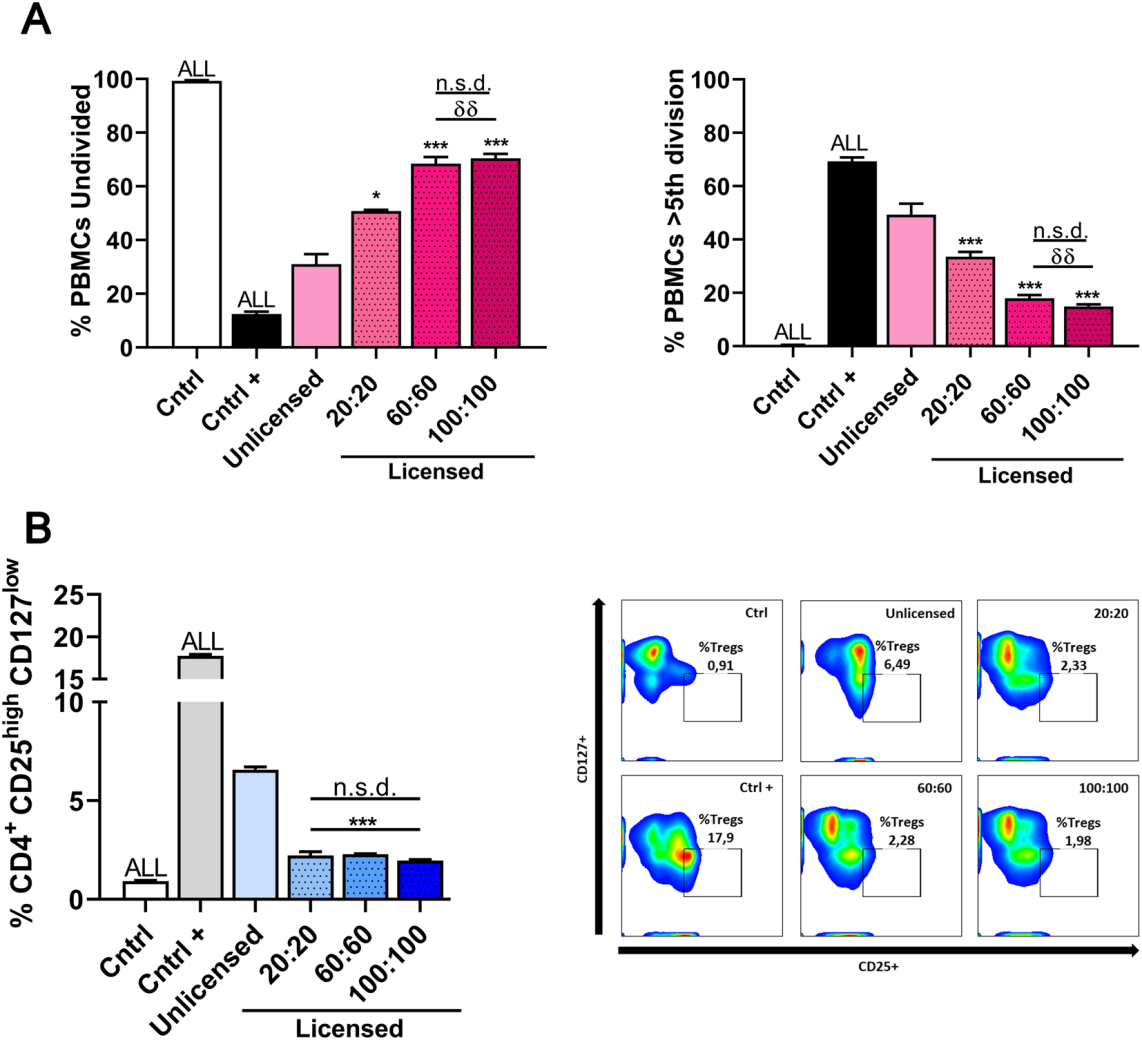
Functional assays to assess MSC-mediated immunomodulation: PBMC proliferation and Treg induction under licensed and unlicensed conditions using different cytokine concentrations (20, 60, and 100 ng/mL) at a 1:1 ratio of IFN-γ and TNF-α. (**A**) Effect of the MSC secretome on PBMC proliferation under licensed and unlicensed conditions. (**B**) Induction of Tregs by the MSC secretome under licensed and unlicensed conditions. Data are presented as mean ± SD (*N* = 3 independent experiments, with 4 replicates per group). Statistical significance: For PBMC proliferation and Treg induction, the control and positive control groups showed significant differences (*p* < 0.001) across all groups. *p < 0.05, ***p < 0.001 compared to the unlicensed group. ^δδ^*p* < 0.01 compared to the 20:20 ng/mL licensed group, with no significant difference (n.s.d.) observed between other licensed groups. Abbreviations: *MSC: mesenchymal stromal cell. PBMCs: peripheral blood mononuclear cells. Treg: regulatory T cells. IFN-γ: interferon γ. TNF-α: tumor necrosis factor α*

The licensed CM demonstrated a significant ability to inhibit PBMC proliferation compared to the unlicensed CM (Fig. 10A). Specifically, the CM licensed with 20 ng/mL of IFN-γ/TNF-α retained 50% of PBMCs in an undivided state and allowed 35% to progress beyond the 5th division. In contrast, the 60 ng/mL dose retained 70% of PBMCs in an undivided state and reduced the percentage of cells beyond the 5th division to 15%, representing a 40% improvement in the retention of undivided PBMCs and a significant reduction in advanced divisions compared to the 20 ng/mL dose (*p* < 0.01). Notably, the 60 ng/mL concentration showed no statistically significant differences compared to the 100 ng/mL dose in either the percentage of undivided PBMCs or the proportion beyond the 5th division, suggesting a plateau effect at higher licensing doses. These findings align with previous results, reinforcing the selection of 60 ng/mL as the optimal dose for generating an immunomodulatory secretome.

We next evaluated the capacity of licensed CM to induce Tregs (CD4 + CD25highCD127low) in human PBMC cultures. After 7 days of incubation, licensed CM at 20, 60, and 100 ng/mL induced less than 3% Tregs, with no statistically significant differences between doses. In contrast, unlicensed CM resulted in approximately 6.5% Treg formation, and the positive control induced 17.9% Tregs (Fig. 10B). These results suggest that, under the tested conditions, the MSC-derived secretome alone is not sufficient to promote significant Treg differentiation. Previous studies have indicated that Treg induction by MSCs may involve additional mechanisms, including direct cell–cell contact and surface molecule engagement [48–50]. To explore this possibility, we performed co-culture experiments using MSCs and PBMCs at varying ratios (Figs. S2, S3). These assays revealed increased Treg induction in the presence of MSCs, supporting the hypothesis that cell-surface interactions may contribute to this effect. Thus, while the MSC secretome exhibits strong immunomodulatory activity, its ability to induce Tregs appears to be limited in the absence of direct cellular interaction.

Taken together, these results support the overall immunosuppressive potential of the licensed CM. The observed inhibition of PBMC proliferation and modulation of immune responses may be partially attributed to increased levels of key immunoregulatory molecules identified in the secretome. IL-1Ra, for instance, antagonizes IL-1β signaling and helps restore immune homeostasis [45]. Galectin-9 interacts with Tim-3 to suppress T cell activation [44], while TGF-β contributes to the suppression of effector T cell responses and the support of regulatory pathways [51]. Although the induction of Tregs was limited under cell-free conditions, the enhanced presence of these molecules suggests that the licensed CM exerts potent paracrine immunomodulatory effects in vitro. Future studies will aim to validate these findings in vivo and expand the immunophenotypic analysis of target immune populations, including FOXP3-expressing Tregs and CD4/CD8 T cell subsets.

## Conclusions

IMIDs represent a significant global health challenge, necessitating the development of innovative therapeutic approaches. While MSC-derived secretomes show promise due to their potent immunomodulatory properties, the lack of standardized protocols for licensing MSCs has hindered their clinical translation. In this study, we developed an optimized licensing protocol using MSCs to produce a secretome with enhanced immunomodulatory efficacy. By combining IFN-γ and TNF-α at a 1:1 ratio and a concentration of 60 ng/mL, alongside 90% cellular confluence and a 48 h CM production period, we achieved a significant improvement in the immunomodulatory potency of the MSC-derived secretome. Licensing MSCs under these conditions increased the secretion of key immunomodulatory cytokines, such as Gal-9 and IL-1Ra, while significantly reducing the levels of growth factors, including HGF and VEGF. To our knowledge, this is the first report describing this functional shift in secretome composition, highlighting a transition from a regenerative to an immunomodulatory profile. The licensing protocol maintained cell viability and prevented the release of cytosolic content, which could otherwise provoke cytotoxic effects. The optimized secretome effectively inhibited the proliferation of PBMCs, achieving a twofold increase in potency compared to suboptimal protocols. However, the induction of Tregs by the MSC secretome was limited, confirming that cell-cell contact is required for effective Treg differentiation. This optimized licensing protocol establishes a reproducible framework for generating a highly immunomodulatory secretome and highlights its potential for the development of innovative MSC-based therapies for IMIDs.

## Electronic supplementary material

Below is the link to the electronic supplementary material.

Supplementary Material 1: Fig. S1. Micrographs at 0, 24, 48, and 72 hours showing MSC morphology under different licensing conditions (0, 20, 60, and 100 ng/mL IFN-γ and TNFγα). *Abbreviations: MSC: mesenchymal stromal cell. IFNγγ: interferon γ. TNF-α: tumor necrosis factor.*

Supplementary Material 2: Fig. S2. Induction of regulatory T cells by MSCs after co-culture for 7 days. Data are presented as mean ± SD. N = 3 independent experiments, with 6 replicates per group. Statistical significance: For Treg induction, the control group showed significant differences (p < 0.001) across all groups. ^$$$^p < 0.001 compared to the positive control group, ^***^p < 0.001 compared to the 1:1 group. Abbreviations: MSCs: mesenchymal stromal cells. *Treg: regulatory T cells*.

Supplementary Material 3: Fig. S3. Flow cytometry histograms highlighting gating selections for the induction of regulatory T cells by MSCs. A) Control. B) Positive control. Abbreviations: MSCs: mesenchymal stromal cells.

Supplementary Material 4

Supplementary Material 5

## Data Availability

The datasets used and/or analyzed during the current study are available from the corresponding author on reasonable request.

## Abbreviations

IMIDs: Immune-mediated inflammatory diseases
MSCs: Mesenchymal stromal cells
Gal-9: Galectin-9
IL-1Ra: Interleukin-1 receptor antagonist
HGF: Hepatocyte growth factor
VEGF: Vascular endothelial growth factor
MSC1: Mesenchymal stromal cell pro-inflammatory phenotype
MSC2: Mesenchymal stromal cell anti-inflammatory phenotype
IFN-γ: Interferon-gamma
TNF-α: Tumor necrosis factor-alpha
JAK/STAT1: Janus knase / signal transducer and activator of transcription 1
NF-κB: Nuclear factor kappa-light-chain-enhancer of activated B cells
IDO: Indoleamine 2,3-dioxygenase
PGE₂: Prostaglandin E2
HLA-G: Human leukocyte antigen G
IL: Interleukin
STAT1: Signal transducer and activator of transcription 1
PD-L1: Programmed death-ligand 1
ICAM-1: Intercellular adhesion molecule 1
VCAM-1: Vascular cell adhesion molecule 1
hTERT-AT-MSCs: Immortalized human adipose tissue-derived mesenchymal stromal cells
AT-MSCs: Adipose tissue–derived mesenchymal stromal cells
PBMC: Peripheral blood mononuclear cells
CM: Conditioned media
LDH: Lactate dehydrogenase
TGF-β: Transforming growth factor beta
SDF-1: Stromal cell–derived factor 1
IL-1β: Interleukin-1 beta
MCP-1: Monocyte chemoattractant protein 1
RANTES: Regulated upon activation, normal T cell expressed and secreted
LIF: Leukemia inhibitory factor
TSG-6: Tumor necrosis factor–stimulated gene 6
Gal-1: Galectin-1
CFSE: Carboxyfluorescein succinimidyl ester
FBS: Fetal bovine serum
P/S: Penicillin/streptomycin
BSA: Bovine serum albumin
Tregs: Regulatory T cells
DAMPs: Damage-associated molecular patterns
