# Supplementary figures and images for: Fine-tuning licensing strategies to boost MSC-based immunomodulatory secretome
Source: Stem Cell Res Ther. 2025 Apr 17;16:183. doi: 10.1186/s13287-025-04315-4 (PMC12004826; doi:10.1186/s13287-025-04315-4)

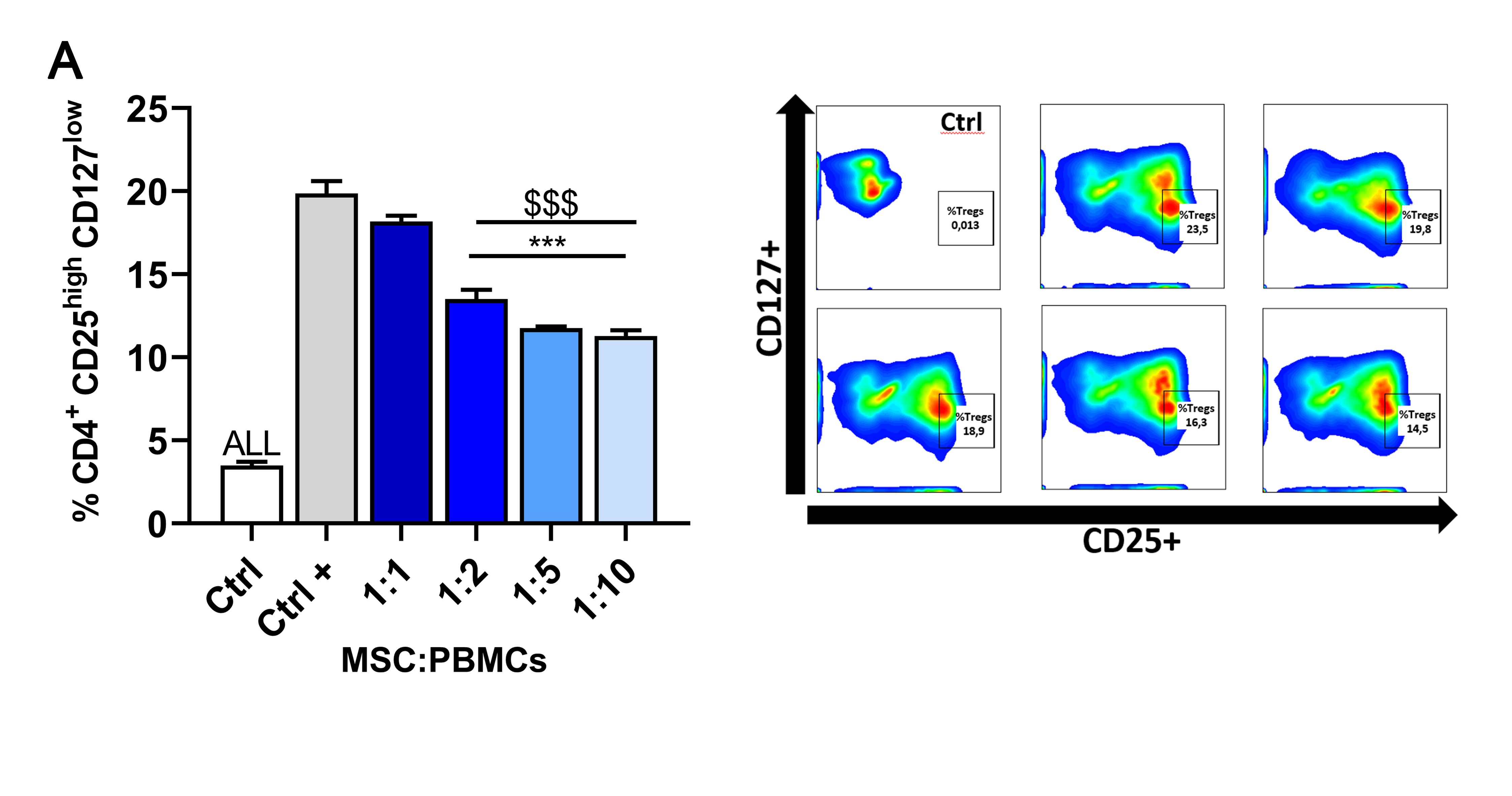

Supplement: Supplementary file 2 — Supplementary Material 2: Fig. S2. Induction of regulatory T cells by MSCs after co-culture for 7 days. Data are presented as mean ± SD. N = 3 independent experiments, with 6 replicates per group. Statistical significance: For Treg induction, the control group showed significant differences (p < 0.001) across all groups. $$$p < 0.001 compared to the positive control group, ***p < 0.001 compared to the 1:1 group. Abbreviations: MSCs: mesenchymal stromal cells. Treg: regulatory T cells. [file 13287_2025_4315_MOESM2_ESM.tif]

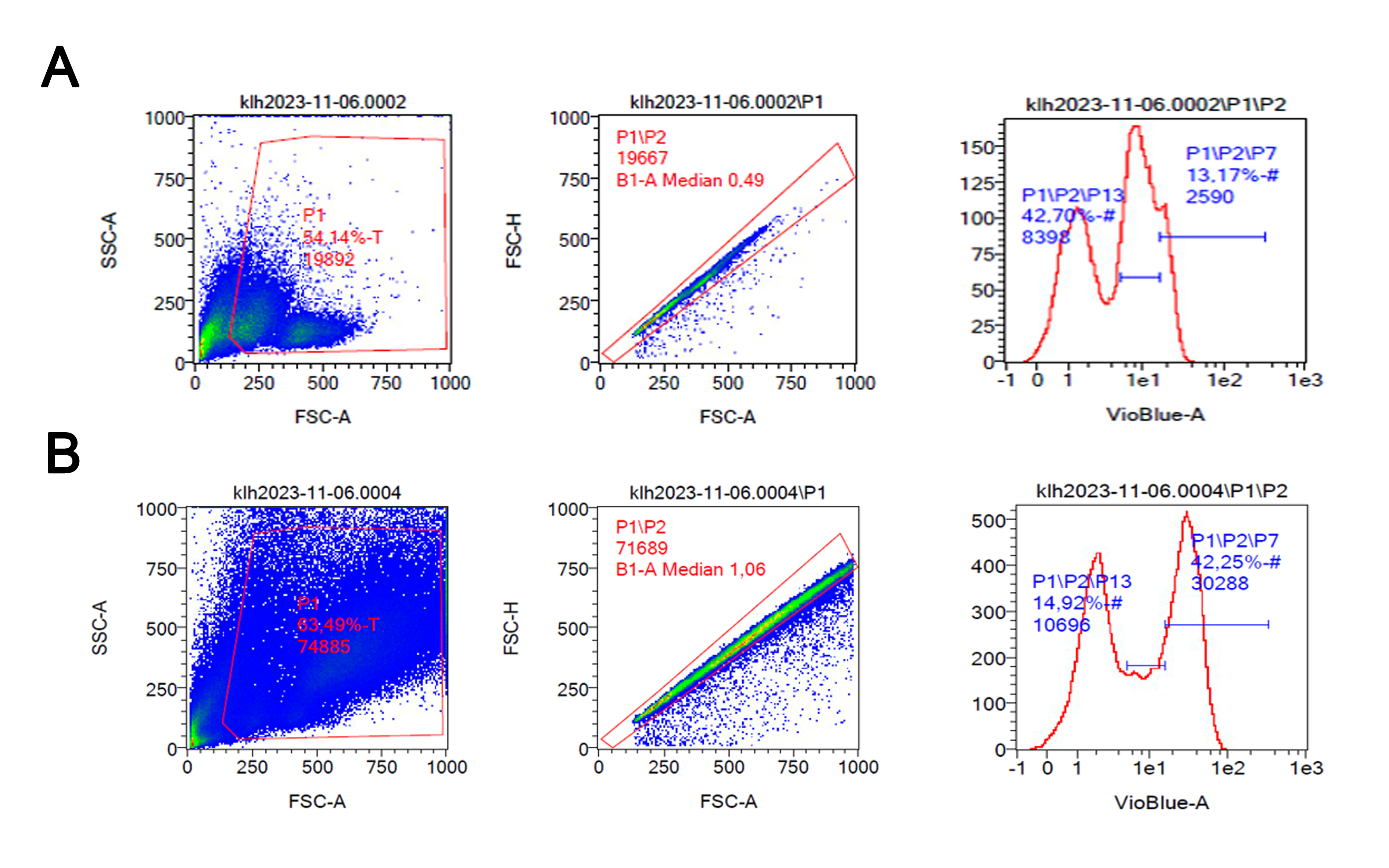

Supplement: Supplementary file 3 — Supplementary Material 3: Fig. S3. Flow cytometry histograms highlighting gating selections for the induction of regulatory T cells by MSCs. A) Control. B) Positive control. Abbreviations: MSCs: mesenchymal stromal cells. [file 13287_2025_4315_MOESM3_ESM.tif]
