# Supplementary material for: Fine-tuning licensing strategies to boost MSC-based immunomodulatory secretome
Source: Stem Cell Res Ther. 2025 Apr 17;16:183. doi: 10.1186/s13287-025-04315-4 (PMC12004826; doi:10.1186/s13287-025-04315-4)
Supplement: Supplementary file 4 — Supplementary Material 4 [file 13287_2025_4315_MOESM4_ESM.pdf]

Material Transfer Agreement

Version 7
20210203

This Material Transfer Agreement ("MTA") is by and between the American Type Culture Collection, a District of Columbia non-profit corporation ("ATCC") and the organization listed below ("Recipient").

Background

ATCC has a nonprofit mission to accept biological materials, maintain them with best practices developed over decades of service as the world's leading biological resource center, and make them available to the research community.

Definitions

"ATCC Original Material" means the materials, including but not limited to genetic, proteomic, and metabolomic data relating to the material, received from ATCC by Recipient as described on an ATCC purchase order.

"ATCC Material" means ATCC Original Material along with its Progeny and Unmodified Derivatives including Unmodified Derivatives existing within Modifications.

"Commercial Use" means the use of the ATCC Materials for commercial benefit, including without limitation:

(i) for sale, license, lease, export, transfer or other distribution for financial purposes or other commercial purposes;

(ii) to provide a service for financial purposes, including but not limited to proficiency testing, preclinical, clinical, bioproduction/manufacturing services or any other fee-for-service use by a CRO, any university core facility, or any other third-party contractor;

(iii) to produce or manufacture products for general sale or ultimately intended for general sale, including use in a commercial manufacturing process such as fermentation, bioproduction or isolation processes;

(iv) in a clinical trial or other testing regulated by a government agency (e.g. FDA, EMEA, EPA, etc.) or in any human;

(v) to collect and commercially exploit data regarding sequences of nucleic acids, proteins or other biological polymers, or relative amounts of biological substances or biological activities; or

(vi) to generate a whole or partial genome sequence and use the foregoing for financial purposes.

"Contributor" means to the entity depositing material with ATCC.

"CRO" means an organization licensed by ATCC to perform services on behalf of Recipient or other clients, including a contract research organization, a contract manufacturing organization, a contract development and manufacturing organization or other similar entities.

"Modifications" means organisms or biological substances created by or on behalf of Recipient which are not Progeny or Unmodified Derivatives, but which contain or incorporate ATCC Material. By way of a nonlimiting example, Modifications result when ATCC Material is modified through molecular biological technology.

"Non-Commercial Use" means use of the ATCC Materials for purposes not constituting Commercial Use.

"**Progeny**" means an unmodified descendant from the ATCC Original Materials, such as plasmid from plasmid, virus from virus, cell from cell, or organism from organism.

"Unmodified Derivative(s)" means substances and sequence data native to and characteristic of ATCC Original Material, including without limitation characteristic nucleic acids, proteins, lipids, carbohydrates, metabolites, membranes, exosomes, organelles, and other native substances, characteristic sequence data of nucleotide, amino acid, or other biological polymerizable monomers, biological polymers expressed according to such characteristic sequence data, antibodies secreted by a hybridoma cell line, or purified or fractionated subsets or lysates of any of the above.

Scope of Use

ATCC MATERIALS ARE NOT FOR USE IN HUMANS. ATCC Materials may only be used by Recipient for research purposes and shall not be used for any Commercial Use without first obtaining a Commercial Use license from ATCC. Use may also be subject to restrictions from a Contributor, a patent owner, or a governmental entity and ATCC makes no representation or warranty regarding the existence or the validity of such restrictions. Notwithstanding anything herein to the contrary, ATCC Materials shall not be used in any manner

that infringes a valid patent in force. Recipient shall have the sole responsibility for identifying and obtaining any third party licenses required.

Transfers

Except as specifically provided in this section, Recipient shall not distribute, sell, transfer or otherwise make available the ATCC Material to any other entity, including its affiliates, without ATCC's prior written approval. Recipient shall ensure that any permitted transfer shall be subject to the permitted transferee agreeing to be bound by terms and conditions of this MTA.

- Core Facility. ATCC Material may not be transferred to or used in any biological material repository or core facility.
- Transfers Within Recipient Organization. Recipient may, for the purposes of the research project make the ATCC Materials available to any Recipient employees solely for use during their employment with Recipient, provided that such employees are made aware of the restrictions against transfer of ATCC Material, including in Modifications, outside of the Recipient organization without ATCC's prior written approval. Recipient accepts responsibility for all violations of such restrictions. Recipient may not (i) use or transfer the ATCC Materials for unrelated projects within Recipient's organization, or (ii) use the ATCC Materials as part of a de facto internal repository or to create a core facility. Recipient shall maintain a record of all such internal transfers and the related projects and shall provide such record to ATCC upon request.
- Non-Commercial Use Research Project Transfers.
    - ATCC Material/Progeny. Any party working on a collaborative research project with Recipient shall obtain ATCC Material or Progeny only from ATCC under an MTA.
    - Modifications and Unmodified Derivatives. Modifications and Unmodified Derivatives may only be made and used by Recipient in its facility. Recipient may transfer Modifications and Unmodified Derivatives to (i) CROs, solely for Non-Commercial Use on Recipient's projects, and (ii) Recipient's research collaborators in a Non- Commercial Use research project, in each case, so long as such transferees agree in writing not to further transfer the Modifications or associated Unmodified Derivatives. Upon completion of any collaborative research project, the Recipient shall require transferee to either return or destroy the Modifications and Unmodified Derivatives and provide an affidavit that no Modifications or Unmodified Derivatives remain in the transferee's possession. Collaborative research projects include, but are not limited to, research sponsored by a for-profit organization carried out at a non-profit organization and by the non-profit organization's personnel. Permitted use hereunder extends only to basic and discovery research related to, directly under, or in direct collaboration with Recipient's research project.
- Transfer of Published Modifications. Recipient may transfer published Modifications for Non-Commercial Use in accordance with the rules of such publication, provided that Recipient notifies ATCC in writing of the transfer.
- Researcher Change of Institution. Should a researcher at Recipient move to another institution, researcher may take Modifications to such institution only if (i) Recipient consents to and notifies ATCC in writing of the transfer and, (ii) Transferee Institution has entered or enters into a material transfer agreement with ATCC.
- Identification of Modifications and Unmodified Derivatives in Transfers. Recipient shall assign and use its own naming scheme when transferring Modifications and Unmodified Derivatives to any transferees and CROs and shall identify such Modifications and Unmodified Derivatives in writing as comprising ATCC Material.
- If the Modifications or Unmodified Derivatives are transferred under any of the conditions described in this Section, Recipient agrees to notify ATCC of the transfer as per the instructions available at www.atcc.org/materialtransfer.

Compliance With Laws

Recipient is solely responsible for and shall ensure compliance with all foreign and domestic, federal, state and local statutes, ordinances and regulations applicable to use of the ATCC Material by Recipient or its transferees, including the U.S. export control laws and related regulations. Recipient is solely responsible for obtaining all permits, licenses or other approvals required by any governmental authority in connection with Recipient's and its transferees' receipt, handling, storage, disposal, transfer and use of the ATCC Materials.

Indemnification; Limitation of Liability

To the extent permitted by law, Recipient shall indemnify, defend and hold harmless ATCC and its Contributors against all third party claims, losses, expenses and damages, including reasonable attorneys' fees (collectively "**Claims**") arising out of or relating to Recipient's and its transferees' use, receipt, handling, storage, transfer, disposal and other activities relating to ATCC Materials, provided that Recipient's liability shall be limited to the extent that any such Claim arises out of ATCC's gross negligence or willful misconduct. All non-monetary settlements of any such Claims are subject to ATCC's prior written consent, which consent shall not be unreasonably withheld. If Recipient is the U.S. federal government or a state institution or a foreign equivalent organization, Recipient shall assume all liability for any and all Claims arising out of or relating to Recipient's and its transferees' use, receipt, handling, storage, transfer, disposal and other activities relating to ATCC Materials to the extent provided under the Federal Tort Claims Act, 28 U.S.C. §§ 2671 et seq. or under equivalent applicable State or foreign law.

ALL ATCC ORIGINAL MATERIALS ARE SUPPLIED "AS IS". ATCC MAKES NO REPRESENTATIONS OR WARRANTIES OF ANY KIND, EITHER EXPRESS OR IMPLIED, AND EXPRESSLY DISCLAIMS WARRANTIES OF MERCHANTABILITY, NONINFRINGEMENT OR FITNESS FOR A PARTICULAR PURPOSE. To the maximum extent permitted by law, in no event will ATCC or its Contributors be liable for any indirect, special, incidental or consequential damages of any kind in connection with or arising out of the MTA or ATCC Materials (whether in contract, tort, negligence, strict liability, statute or otherwise) even if ATCC has been advised of the possibility of such damages. In no event shall ATCC's cumulative liability to the Recipient exceed the fees paid by Recipient under this MTA and the applicable ATCC purchase order for the twelve (12) month period preceding the date of the event giving rise to the first such claim. Recipient agrees that the limitations of liability set forth in this MTA shall apply even if a limited remedy provided hereunder fails of its essential purpose.

The provisions of this *Indemnification; Limitation of Liability* section shall survive termination or expiration of this MTA.

Property Rights

ATCC and/or its Contributors shall retain ownership of all right, title and interest in the ATCC Materials, including such ATCC Materials contained or incorporated in Modifications. Recipient retains ownership of: (a) Modifications (except that, as between the parties, ATCC retains ownership rights to ATCC Material included therein and use of ATCC Materials remains subject to Scope of Use, above) and (b) those substances created through the use of ATCC Material, but which do not contain ATCC Material.

Recipient shall cite ATCC as the provider of ATCC Materials and give appropriate credit to ATCC in any publication containing information derived from ATCC Materials.

Recipient expressly acknowledges that all ATCC trademarks are the exclusive property of ATCC and ATCC retains all right, title and interest in and to its trademarks, service marks, trade-names, logos, catalog numbers and ATCC-specific designations of ATCC Materials. Except as mandated by this *Property Rights* section, Recipient shall not to use the foregoing in any way without ATCC's prior written agreement.

Miscellaneous

Any disputes arising under this MTA shall be governed by the laws of the State of New York without regard to its conflict of laws principles, and Recipient hereby expressly consents to, submits to and waives any objection to the sole and exclusive jurisdiction and venue of New York courts. If Recipient is a Federal or State non-profit organization or foreign public organization, then any disputes arising under this MTA shall be tried exclusively in a court of competent jurisdiction.

Recipient agrees that any breach of this MTA, including but not limited to any breach of the Scope of Use provisions of this MTA, will entitle ATCC to immediately cease further shipments of ATCC Material without notice to Recipient and ATCC shall have the right to immediately terminate this MTA. Recipient

acknowledges that any breach may create such irreparable injury as to entitle ATCC to seek preliminary or permanent injunctive relief in addition to all other equitable and legal remedies available under applicable laws.

Recipient may not assign or otherwise transfer this MTA or any rights or obligations under this MTA, whether by operation of law or otherwise without the prior written consent of ATCC and any such attempted assignment or transfer will be void and of no force or effect. This MTA shall be binding on all permitted successors and assigns. This MTA, including all documents incorporated herein by reference, constitutes the entire agreement between ATCC and Recipient with respect to the ATCC Material

and supersedes all previous agreements between ATCC and Recipient relating to the ATCC Material. No term or provision contained herein shall be deemed waived or modified and no breach excused unless such waiver or consent shall be in writing and signed by the parties. If any provision of this MTA is for any reason found to be unenforceable, the remainder of this MTA will continue in full force and effect. The provisions of this MTA which by their nature or implication are intended to survive termination or expiration shall so survive. None of the provisions of this MTA are intended to create, nor shall be deemed or construed to create, any relationship between ATCC or Recipient other than that of independent entities contracting with each other hereunder solely for the purpose of effecting the provisions of this MTA. The undersigned represent that they have full authority to enter into this MTA and to bind the parties for and on behalf of the legal entities set forth below.

AGREEMENT TO THIS MTA witnessed by the following signature as of this effective date: 17/MAR/2021

Recipient Organization: UNIVERSIDAD DEL PAIS VASCO/EUSKAL HERRIKO UNIBERTSITATEA

Organization tax ID: Q4818001B

Signed:

By: GUILLERMO QUINDÓS ANDRÉS

Its: VICE-CHANCELLOR FOR SCIENTIFIC-SOCIAL DEVELOPMENT AND TRANSFER

Email: vrdesarrollo-transferencia@ehu.eus

Address: Barrio Sarriena - s/n - 48940 Leioa (Bizkaia), SPAIN

Any correspondence concerning this MTA should be addressed to: ATCC, ATTN: Contracts, 10801 University Boulevard, Manassas, VA 20110. Otherwise, please contact us at **contracts@atcc.org**.

14952802L GUILLERMO QUINDOS (R: Q4818001B)

Firmado digitalmente por 14952802L GUILLERMO QUINDOS (R: Q4818001B)
Fecha: 2021.03.17 14:24:47 +01'00'
