# Supplementary material for: Fine-tuning licensing strategies to boost MSC-based immunomodulatory secretome
Source: Stem Cell Res Ther. 2025 Apr 17;16:183. doi: 10.1186/s13287-025-04315-4 (PMC12004826; doi:10.1186/s13287-025-04315-4)
Supplement: Supplementary file 5 — Supplementary Material 5 [file 13287_2025_4315_MOESM5_ESM.pdf]

ACUERDO DE TRANSFERENCIA DE MATERIALES

V.7.1

El presente Acuerdo de Transferencia de Materiales ("**MTA**") se celebra por y entre la American Type Culture Collection, una sociedad sin ánimo de lucro del Distrito de Columbia (en adelante, la "**ATCC**") y la organización que se indica a continuación (en adelante, el "**Adquirente**").

Antecedentes

La ATCC tiene la misión sin ánimo de lucro de aceptar materiales biológicos, mantenerlos con las mejores prácticas desarrolladas durante décadas de servicio como el centro de recursos biológicos líder en el mundo y ponerlos a disposición de la comunidad de investigadores.

Definiciones

"**Material Original ATCC**" significa los materiales, que incluyen, entre otros, datos genéticos, proteómicos y metabolómicos relacionados con el material, adquiridos de la ATCC por el Adquirente, tal como se describe en la Orden de Venta ATCC.

"**Material ATCC**" significa Material Original ATCC junto con su Progenie y Derivados No Modificados que incluyen Derivados No Modificados existentes dentro de Modificaciones.

"**Uso Comercial**" significa el uso de los Materiales ATCC para beneficio comercial, que incluye, entre otros:

(i) La venta, licencia, arrendamiento, exportación, transferencia u otro tipo de distribución para obtener ganancias financieras u otros propósitos comerciales.
(ii) Proporcionar un servicio para obtener ganancias financieras, que incluye, entre otros, pruebas de aptitud, servicios preclínicos, clínicos, de bioproducción/fabricación o cualquier otro uso de pago por servicio por parte de una CRO, cualquier instalación central de universidad o cualquier otro contratista externo.
(iii) Producir o fabricar productos para la venta general o, en última instancia, destinados a la venta general, que incluye el uso en un proceso de fabricación comercial, tal como procesos de fermentación, bioproducción o aislamiento.
(iv) En un ensayo clínico u otras pruebas reguladas por una agencia gubernamental (por ejemplo, FDA, EMEA, EPA, etc.) o cualquier ser humano.
(v) Recopilar y explotar comercialmente datos sobre secuencias de ácidos nucleicos, proteínas u otros polímeros biológicos, o cantidades relativas de sustancias biológicas o actividades biológicas.
(vi) Generar una secuencia de genoma total o parcial y usarla para obtener ganancias financieras.

"**Contribuyente**" significa la entidad que deposita material con la ATCC.

"**CRO**" significa una organización que presta servicios en nombre del Adquirente u otros clientes, que incluye una organización de investigación por contrato, una organización de fabricación por contrato, una organización de fabricación y desarrollo por contrato u otras entidades similares. Cualquier CRO que brinde servicios por una tarifa debe consultar con ATCC sobre su necesidad de una licencia.

"**Modificaciones**" significa organismos o sustancias biológicas creadas por o en nombre del Adquirente que no son Progenie ni Derivados No Modificados, pero que contienen o incorporan Material ATCC. A modo de ejemplo no limitante, las Modificaciones se producen cuando el Material ATCC se modifica mediante tecnología de biología molecular.

"**Uso No Comercial**" significa el uso de los Materiales ATCC para fines que no constituyen un Uso Comercial.

"**Progenie**" significa un descendiente no modificado de los Materiales Originales ATCC, tales como un plásmido de un plásmido, un virus de un virus, una célula de una célula o un organismo de un organismo.

"**Derivado(s) No Modificado(s)**" significa sustancias y datos de secuencia nativos y característicos del Material Original ATCC, que incluyen, entre otros, ácidos nucleicos, proteínas, lípidos, carbohidratos, metabolitos, membranas, exosomas, orgánulos característicos y otras sustancias nativas, datos de secuencia característicos de nucleótidos, aminoácidos u otros monómeros biológicos polimerizables, polímeros biológicos expresados de acuerdo con dichos datos de secuencia característicos, anticuerpos secretados por una línea celular hibridoma, o subconjuntos o lisados purificados o fraccionados de cualquiera de lo mencionado anteriormente.

Ámbito de Uso

LOS MATERIALES ATCC NO SON PARA USO EN HUMANOS. Los Materiales ATCC solo pueden ser usados por el Adquirente con fines de investigación y no se usarán para ningún Uso Comercial sin antes obtener una licencia de Uso Comercial de la ATCC**.** El uso también puede estar sujeto a restricciones de un Contribuyente, un propietario de patente o una entidad gubernamental y la ATCC no representa ni garantiza la existencia o validez de dichas restricciones. Sin perjuicio de lo establecido en el presente documento que indique lo contrario, los Materiales ATCC no se usarán de ninguna manera que infrinja una patente válida en vigencia. El Adquirente tendrá la responsabilidad exclusiva de identificar y obtener cualquier licencia de terceros requerida.

Transferencias

Salvo que se disponga específicamente en esta sección, el Adquirente no distribuirá, venderá, transferirá ni pondrá a disposición de ninguna otra forma el Material ATCC a ninguna otra entidad, incluidas sus filiales, sin la aprobación previa por escrito de la ATCC. El Adquirente se

asegurará de que cualquier transferencia autorizada esté sujeta al acuerdo por parte del Beneficiario autorizado de estar sujeto a los términos y condiciones del presente MTA.

- Instalación Central. El Material ATCC no se puede transferir ni usar en ningún depósito o instalación central de material biológico.
- Transferencias Dentro de la Organización del Adquirente. El Adquirente puede, a los efectos del proyecto de investigación, poner los Materiales ATCC a disposición de los empleados del Adquirente únicamente para su uso durante su tiempo como empleados del Adquirente, siempre que dichos empleados conozcan las restricciones de la transferencia de Material ATCC, incluso en Modificaciones, fuera de la organización del Adquirente sin la aprobación previa por escrito de la ATCC. El Adquirente acepta la responsabilidad de todas las violaciones a dichas restricciones. El Adquirente no puede (i) usar ni transferir los Materiales ATCC para proyectos no relacionados dentro de la organización del Adquirente, ni (ii) usar los Materiales ATCC como parte de un depósito interno *de facto* o para crear una instalación central. El Adquirente mantendrá un registro de todas esas transferencias internas y los proyectos relacionados y proporcionará dicho registro a la ATCC cuando lo solicite.
- Transferencias de Proyectos de Investigación de Uso No Comercial.
    - Material Original ATCC/Progenie. Cualquier parte que trabaje en un proyecto de investigación colaborativa con el Adquirente deberá obtener Material l Original ATCC o Progenie únicamente de la ATCC.
    - Modificaciones y Derivados No Modificados. Las Modificaciones y los Derivados No Modificados solo se pueden fabricar y usar por el Adquirente en su instalación. El Adquirente puede transferir las Modificaciones y los Derivados No Modificados a (i) las CRO, únicamente para Uso No Comercial en los proyectos del Adquirente, y (ii) los colaboradores de investigación del Adquirente en un proyecto de investigación de Uso No Comercial, en cada caso, siempre que dichos Beneficiarios acuerden por escrito no seguir transfiriendo las Modificaciones o los Derivados No Modificados asociados. Una vez finalizado cualquier proyecto de investigación colaborativa, el Beneficiario deberá exigir al cesionario que devuelva o certifique la destrucción de las Modificaciones y los Derivados No Modificados. Los proyectos de investigación colaborativa incluyen, entre otros, investigaciones patrocinadas por una organización con fines de lucro realizadas en una organización sin fines de lucro y por el personal de la organización sin fines de lucro. El uso permitido en el presente documento se extiende solo a la investigación básica y de descubrimiento relacionada, directamente bajo, o en colaboración directa con el proyecto de investigación del Adquirente.
- Transferencia de Modificaciones Publicadas. El Adquirente puede transferir Modificaciones Publicadas para Uso No Comercial de acuerdo con las reglas de la publicación, siempre que el Adquirente notifique sobre la transferencia por escrito a la ATCC.
- Cambio de Institución del Investigador. Si un investigador del Adquirente se muda a otra institución, el investigador puede llevar Modificaciones a dicha institución solo si (i) el Adquirente permite la transferencia y la notifica por escrito a la ATCC y (ii) la Institución del Beneficiario ha celebrado o celebra un acuerdo de transferencia de materiales con la ATCC.
- Identificación de Modificaciones y Derivados No Modificados en las Transferencias. El Adquirente deberá asignar y usar su propio sistema de nomenclatura al transferir Modificaciones y Derivados No Modificados a cualquier Beneficiario y a las CRO y deberá identificar dichas Modificaciones y Derivados No Modificados por escrito como si constituyeran Material ATCC.
- Si las Modificaciones o los Derivados No Modificados se transfieren bajo cualquiera de las condiciones descritas en esta Sección, el Adquirente acepta notificar a la ATCC de la transferencia según las instrucciones disponibles en www.atcc.org/transfer.

Cumplimiento de las leyes

El Adquirente es el único responsable y deberá garantizar el cumplimiento de todos los estatutos, ordenanzas y normas extranjeras y nacionales, federales, estatales y locales aplicables al uso del Material ATCC por parte del Adquirente o sus Beneficiarios, incluidas las leyes de control de exportaciones de los Estados Unidos y normas relacionadas. El Adquirente es el único responsable de obtener todos los permisos, licencias u otras aprobaciones requeridas por cualquier autoridad gubernamental en relación con la recepción, manejo, almacenamiento, eliminación, transferencia y uso de los Materiales ATCC por parte del Adquirente o sus Beneficiarios.

Indemnización; Limitación de la Responsabilidad

En la medida permitida por la ley, el Adquirente indemnizará, defenderá y mantendrá indemne a la ATCC y sus Contribuyentes contra toda reclamación, pérdida, costo y daño a terceros, incluyendo honorarios legales razonables (colectivamente "**Reclamaciones**") que surjan de o estén relacionadas con el uso, recepción, manejo, almacenamiento, transferencia, eliminación y otras actividades relacionadas con los Materiales ATCC por parte del Adquirente o sus Beneficiarios, siempre que la responsabilidad del Adquirente esté limitada en la medida en que dicha Reclamación surja de una negligencia grave o conducta impropia deliberada de la ATCC. Todos los acuerdos de carácter no monetario sobre cualquiera de dichas Reclamaciones están sujetos al consentimiento previo por escrito de la ATCC, no debiendo dicho consentimiento ser denegado de forma arbitraria. Si el Adquirente es el gobierno federal de los Estados Unidos o una institución estatal o una organización extranjera equivalente, el Adquirente asumirá la responsabilidad por todas y cada una de las Reclamaciones que surjan de o estén relacionadas con el uso, recepción, manejo, almacenamiento, transferencia, eliminación y otras actividades relacionadas con los Materiales ATCC por parte del Adquirente y sus Beneficiarios en la medida prevista bajo Federal Tort Claims Act (Ley de Reclamaciones Extracontractuales), 28 U.S.C. §§ 2671 et seq. o bajo una ley Estatal o extranjera equivalente aplicable.

TODOS LOS MATERIALES ORIGINALES ATCC SE SUMINISTRAN "TAL CUAL". ATCC NO HACE REPRESENTACIONES NI GARANTÍAS DE NINGÚN TIPO, YA SEAN EXPRESAS O IMPLÍCITAS, Y NIEGA EXPRESAMENTE CUALQUIER GARANTÍA DE COMERCIABILIDAD, NO INFRACCIÓN O IDONEIDAD PARA UN PROPÓSITO PARTICULAR. En la medida máxima permitida por la ley, en ningún caso la ATCC o sus Contribuyentes serán responsables de ningún tipo de daño indirecto, especial, incidental o consecuente en relación con o que surja del MTA o los Materiales ATCC (ya sea por contrato, extracontractual, negligencia, responsabilidad objetiva, estatuto o de otro tipo), incluso si se ha informado a la ATCC de la posibilidad de dichos daños. En ningún caso la responsabilidad acumulada de la ATCC con el Adquirente excederá los honorarios pagados por el Adquirente bajo el presente MTA y la Orden de Venta ATCC aplicable durante el período de doce (12) meses anteriores a la fecha del evento que dio lugar a la primera de dichas reclamaciones. El Adquirente acepta que las limitaciones de responsabilidad establecidas en el presente MTA se aplicarán incluso si un recurso limitado provisto en el presente documento no cumple su propósito esencial.

Las disposiciones de esta sección de Indemnización permanecerán vigentes al término o vencimiento del presente Acuerdo.

Derechos de propiedad

La ATCC y/o sus Contribuyentes conservarán la propiedad de todos los derechos, títulos e intereses de los Materiales ATCC, incluso los Materiales ATCC contenidos o incorporados en las Modificaciones. El Adquirente conserva la propiedad de: (a) las Modificaciones (excepto que, al igual que entre las partes, la ATCC conserva los derechos de propiedad sobre el Material ATCC incorporado en las mismas y el uso de los Materiales ATCC permanece sujeto al Ámbito de Uso mencionado anteriormente) y (b) aquellas sustancias creadas mediante el uso de Material ATCC, pero que no contienen Material ATCC.

En cualquier publicación que contenga información derivada de los Materiales ATCC, el Adquirente reconoce citar a la ATCC como el proveedor de los Materiales ATCC y otorgarle el crédito correspondiente.

El Adquirente reconoce expresamente que todas las marcas registradas de la ATCC son propiedad exclusiva de la ATCC y que la ATCC conserva todos los derechos, títulos e intereses sobre sus marcas registradas, marcas de servicio, nombres comerciales, logotipos, números de catálogo y designaciones específicas de la ATCC de los Materiales ATCC. Con excepción de lo establecido en esta sección de Derechos de propiedad, el Adquirente no usará lo mencionado anteriormente de ninguna manera sin el acuerdo previo por escrito de la ATCC.

Disposiciones Varias

Cualquier disputa que surja bajo el presente MTA se regirá por las leyes del Estado de Nueva York sin tener en cuenta sus principios de conflicto de leyes, y el Adquirente por la presente otorga expresamente su consentimiento, se somete y renuncia a cualquier objeción a la jurisdicción única y exclusiva de dichas cortes. Si el Adquirente es una organización sin fines de lucro Federal o Estatal o una organización pública extranjera, entonces cualquier disputa que surja bajo el presente MTA se juzgará exclusivamente en una corte de jurisdicción competente.

El Adquirente acepta que cualquier incumplimiento del presente MTA, incluido, entre otros, cualquier incumplimiento de las disposiciones del Ámbito de Uso del presente MTA, dará derecho a la ATCC a suspender inmediatamente cualquier envío de Material ATCC sin previo aviso al Adquirente y la ATCC tendrá derecho a rescindir inmediatamente el presente MTA. El Adquirente reconoce que cualquier incumplimiento puede crear un daño irreparable que dé derecho a la ATCC a buscar medidas cautelares preliminares o permanentes además de todos los demás recursos legales y equitativos disponibles bajo las leyes aplicables.

El Adquirente no podrá ceder o de forma alguna transferir el presente MTA, así como ningún derecho u obligación bajo el mismo, ya sea por disposición de la ley o de cualquier otra manera sin el consentimiento previo por escrito de la ATCC y cualquier intento de cesión o transferencia será nulo y no tendrá efecto. El presente MTA será vinculante para todos los sucesores y cesionarios autorizados. El presente MTA, incluidos todos los documentos incorporados aquí como referencia, constituyen el acuerdo completo entre la ATCC y el Adquirente con respecto al Material ATCC y reemplazan todos los acuerdos anteriores entre la ATCC y el Adquirente en relación con el Material ATCC. Ningún término o disposición contenido en el presente documento será considerado como cancelado o modificado y ningún incumplimiento será justificado a menos que dicha cancelación o consentimiento sea por escrito y esté firmado por las partes. Si por alguna razón alguna disposición del presente MTA es considerada inaplicable, el resto del presente MTA permanecerá en pleno vigor y efecto. Las disposiciones del presente MTA, que por su naturaleza o implicación están destinadas a permanecer vigentes al término o vencimiento, permanecerán vigentes. Ninguna de las disposiciones del presente MTA pretende crear, ni se considerará ni interpretará que puede crear, alguna relación entre la ATCC o el Adquirente, diferente a la de entidades independientes con un contrato entre sí bajo el presente documento, con el único propósito de hacer efectivas las disposiciones del presente MTA. Los abajo firmantes declaran que tienen plena autoridad para celebrar este Acuerdo y vincular a las partes en nombre de las entidades legales indicadas a continuación.

ACEPTACIÓN DEL PRESENTE MTA como lo atestigua la siguiente firma a partir de la presente Fecha de vigencia: 12/11/2024

ORGANIZACIÓN DEL ADQUIRENTE: Universidad del País Vasco - Euskal Herriko Unibertsitatea

Nº identificación fiscal (NIF): Q4818001B

Firmado: 30590971M INMACULADA AROSTEGUI (R: Q4818001B) Firmado digitalmente por 30590971M INMACULADA AROSTEGUI (R: Q4818001B) Fecha: 2024.11.14 20:06:00 +01'00'

Por: Inmaculada Arostegui Madariaga (1)

Cargo: Ikerketaren arloko errektoreordea - Vicerrectora de Investigación

Correo electrónico: iproperty.otri@ehu.eus

Dirección: Bº Sarriena s/n, 48940 - Leioa

(1) Zientzia eta Gizarte Garapenaren eta Transferentziaren arloko errektoreorde Kargua hutsik geratzeagatik, gobernu-taldearen ordezkapen-araubidearen UPV/EHUko errektorearen, 2021eko urtarrilaren 28ko Ebazpenarekin bat etorrita (2021eko otsailaren 12ko EHAA).
Por vacancia del Vicerrectorado de Desarrollo Científico-social y Transferencia conforme a la Resolución de 28 de enero de 2021, de la Rectora de la UPV/EHU, sobre régimen de suplencias de su equipo de gobierno (BOPV de 12 de febrero de 2021).

Toda correspondencia relacionada con el presente MTA debe ser dirigida a: **American Type Culture Collection, Attention: Contracts, 10801 University Blvd, Manassas, VA 20110.** O contáctese con nosotros al correo electrónico contracts@atcc.org.
